# Supplementary material for: Concomitant deletion of Ptpn6 and Ptpn11 in T cells fails to improve anticancer responses
Source: EMBO Rep. 2022 Oct 4;23(11):e55399. doi: 10.15252/embr.202255399 (PMC9638855; doi:10.15252/embr.202255399)
Supplement: Supplementary file 3 — Source Data for Expanded View [file EMBR-23-e55399-s008.zip › Fig EV1.pdf]

Fig EV1 A - Spleen

	Ptpn6$^{fl/fl}$	CD4cre Ptpn6$^{fl/fl}$
**Cellularity**	78250000	66000000
	102875000	86875000
	56000000	34750000
	36000000	44000000
	43750000	40375000
	38250000	43625000
	17250000	35125000
	38750000	40625000

	Ptpn6$^{fl/fl}$	CD4cre Ptpn6$^{fl/fl}$
**CD4 numbers**	17296703	11020300
	17710750	9753478
	10450944	5303823
	7476300	5099600
	6601437.5	4582481.75
	5703457.5	5553680.625
	3219384.75	3862907
	5365906.25	4500803.125

	Ptpn6$^{fl/fl}$	CD4cre Ptpn6$^{fl/fl}$
**CD8 numbers**	9142543	8186427
	9965280	6197112
	5583424	3621645
	3705336	4282256
	3923062.5	3434232.9
	4055609.25	4023621
	2123820	1972936.125
	2077263.5	2509390

	Ptpn6$^{fl/fl}$	CD4cre Ptpn6$^{fl/fl}$
**CD4 CD44exp**	22.4	37.7
	32.4	45.1
	27.3	32.8
	26.5	34.9
	25	35.1
	30	34.4
	33.1	39.8
	30.6	33.1

	Ptpn6$^{fl/fl}$	CD4cre Ptpn6$^{fl/fl}$
**CD8 CD44exp**	42.8	59.9
	33.3	58.2
	48	62.7
	39.7	69.3
	29	58.2
	27.9	56.2
	35.7	67.8
	46.1	67.1

	Ptpn6$^{fl/fl}$	CD4cre Ptpn6$^{fl/fl}$
**Treg numbers**	2300461	1774268
	2514926	1570310
	1463132	859219
	1091540	820512
	1042912.5	868224
	794414.25	1131807
	442531.5	715074.75

	Ptpn6$^{fl/fl}$	CD4cre Ptpn6$^{fl/fl}$
**Treg freq**	13.3	16.1
	14.2	16.1
	14	16.2
	14.6	14.8
	13.7	16.8
	12.9	18.4
	12.7	17.4

Fig EV1 B - LNs

	Ptpn6$^{fl/fl}$	CD4cre Ptpn6$^{fl/fl}$
**Cellularity**	5437500	3162500
	4812500	3337500
	5137500	2075000
	2812500	1637500
	2137500	1975000
	1900000	1400000
	2087500	2175000
		2412500

	Ptpn6$^{fl/fl}$	CD4cre Ptpn6$^{fl/fl}$
**CD4 numbers**	2521847	1199125
	2346397	1156003
	2512484	760840
	1248750	491250
	780187.5	673475
	729600	449400
	843350	856950
		890212.5

	Ptpn6$^{fl/fl}$	CD4cre Ptpn6$^{fl/fl}$
**CD8 numbers**	1889803	1213552
	1432200	1172424
	1545766	608058
	791100	499293.4
	661428	591014.8
	586074	399546
	411803.2125	495869.55
		602268.5625

	Ptpn6$^{fl/fl}$	CD4cre Ptpn6$^{fl/fl}$
**CD4 CD44exp**	7.6	7.7
	6.6	10.3
	7.8	8.8
	6.7	8.8
	6.5	10.2
	9.9	10.2
	10.2	10.9
	10.3	11.5

	Ptpn6$^{fl/fl}$	CD4cre Ptpn6$^{fl/fl}$
**CD8 CD44exp**	31.9	50.6
	23.5	55.3
	37.6	58
	29	64.6
	25.6	56.9
	26.1	58.2
	29.8	56.6
	41.2	66.5

Fig EV1 C - Thymus

	Ptpn6/11$^{fl/fl}$	CD4cre Ptpn6/11$^{fl/fl}$
**Cellularity**	80500000	77000000
	77125000	86125000
	95500000	143750000
	107625000	161250000
	114250000	171250000
	50500000	166250000
	69125000	54750000
	84875000	101125000
	62625000	73000000
	59000000	74000000
		60875000
		55625000

	Ptpn6/11$^{fl/fl}$	CD4cre Ptpn6/11$^{fl/fl}$
**CD4- CD8- numbers**	1288000	1031800
	987200	1843075
	1785850	2458125
	2163263	2934750
	1919400	2894125
	1090800	2743125
	1859463	1297575
	2851800	3468588
	1509263	2620700
	1268500	2353200
		1375775
		1051313

	Ptpn6/11$^{fl/fl}$	CD4cre Ptpn6/11$^{fl/fl}$
**CD4+ CD8+ numbers**	71162000	69839000
	68487000	76565125
	82703000	128943750
	93633750	142061250
	99283250	151213750
	44137000	149458750
	60968250	49713000
	73077375	89799000
	55360500	64240000
	52097000	66156000
		55883250
		50563125

	Ptpn6/11$^{fl/fl}$	CD4cre Ptpn6/11$^{fl/fl}$
**CD4+ numbers**	3336000	2266480
	5428440	1829520
	5114850	3024000
	4405451	2891490
	4519834	4406700
	3177056	1228920
	3471644	2039788
	4298172	3524965
	3190982	2500900
	3351194	2466198
		1965325
		2314723

	Ptpn6/11$^{fl/fl}$	CD4cre Ptpn6/11$^{fl/fl}$
**CD8+ numbers**	1079360	324786
	977667	671052
	1584192	791516
	1514284	1134136
	1932836	1324722
	825236	900161
	660946	230541
	941773	363413
	686533	312732
	714655	252414
		224909
		286358

Fig EV1 D - Tregs

	Ptpn6/11$^{fl/fl}$	CD4cre Ptpn6/11$^{fl/fl}$
**Treg number**	745051	1618570
	982674	977421
	1118003	2070470
	1080361	1245408
	905389	1098961
	2395120	1461380
	2060984	1554525
	1622600	2720200
	1214010	2439747
	1117783	1401680
		1749420
		1386725

	Ptpn6/11$^{fl/fl}$	CD4cre Ptpn6/11$^{fl/fl}$
**Treg freq**	15.1	26.6
	13.4	22.1
	14.3	19.8
	11.8	18.7
	10.6	20.4
	15.3	23
	14.6	22.5
	14.5	21.2
	13.7	21.8
	11.5	13
		14.6
		11.9

Fig EV1 E - LNs

	Ptpn6/11$^{fl/fl}$	CD4cre Ptpn6/11$^{fl/fl}$
**Cellularity**	2443750	490000
	3962500	1581250
	4431250	3818750
	4537500	1625000
	4562500	1618750
	4687500	1606250
	4037500	4237500
	6650000	4912500
	4500000	2500000
	5025000	4237500
		2087500
		2387500

	Ptpn6/11$^{fl/fl}$	CD4cre Ptpn6/11$^{fl/fl}$
**CD4 numbers**	954663	113011
	1743916	432089
	1699624	1037173
	1707325	414255
	1810281	448018
	1898813	397007
	1598729	819499
	2621510	1160136
	1832481	739870
	2281104	1251863
		555409
		635553

	Ptpn6/11$^{fl/fl}$	CD4cre Ptpn6/11$^{fl/fl}$
**CD8 numbers**	1030285	136406
	1527540	622560
	1504533	1517793
	1642457	676494
	1621558	493144
	2004750	612585
	1458515	1730790
	2402259	1460162
	1539090	945990
	1707857	1248876
		690203
		907642

	Ptpn6/11$^{fl/fl}$	CD4cre Ptpn6/11$^{fl/fl}$
**CD4 CD44exp**	19	37
	14.9	33
	15.2	31.8
	16.2	25.9
	14.8	30.9
	14.8	30.8
	11.8	29.8
	10.3	26.6
	13.6	25.8
	11.5	25.7
		27.2
		28.7

	Ptpn6/11$^{fl/fl}$	CD4cre Ptpn6/11$^{fl/fl}$
**CD8 CD44exp**	36.3	73.4
	24.4	67.8
	33.1	73.3
	40.7	67
	28.5	68.9
	37.5	72.8
	28.9	70.6
	25.1	67
	32.8	59.7
	24.9	60.2
		81.4
		80.6

Fig EV1 F - Survival CD4cre Ptpn6/11^{Bgn}

Days	Ptpn6/11$^{fl/fl}$	CD4cre Ptpn6/11$^{fl/fl}$
**11**		
**12**		
**12**		
**12**		
**13**		1
**13**		1
**13**		1
**13**		
**14**		1
**14**		1
**14**		1
**15**	1	
**16**	1	1
**16**	1	1
**16**	1	1
**17**	1	1
**17**	1	
**17**	1	
**18**	1	
**19**	1	
**20**	1	
**21**	1	

Fig EV1 G - PD1 exp TILs

	CD4cre Ptpn6/11$^{wt/wt}$ Iso	CD4cre Ptpn6/11$^{fl/fl}$ Iso
**CD8+ PD-1+**	37.9	17.9
	43.6	25.2
	44.3	56.4
	43.7	29.4
	52.1	39.3
	21.7	38.3
	44.4	34.7
	42.3	48.3
	21.7	41
	39	35.2
	40	55
	74.4	25.4
	55.3	39.8
	45.2	19.4

Fig EV1 H - Tumor CD4 cells cytokines

	CD4cre Ptpn6/11$^{wt/wt}$ Iso	CD4cre Ptpn6/11$^{wt/wt}$ aPD1	CD4cre Ptpn6/11$^{fl/fl}$ Iso	CD4cre Ptpn6/11$^{fl/fl}$ aPD1
**IFN-y+**	3.61	8.38	3.26	5.75
	2.76	4.21	1.08	3.4
	7.94	9.59	1.47	2.62
	4.89	23.7	3.72	1.39
	3.23	28.4	2.12	38.6
	8.17	36.3	27.1	44.3
	6.06	5.76	33.8	27.3
	39.5	5.41	28	31.4
	27.4	9.91	32.4	44.4
	36.9		26	5
	3.46		36.5	3.81
	8.28		9.32	
	3.78		10.12	
	6.88		5.72	
			4.57	
**TNF-a+**	16.97	15.84	39.61	8.05
	18.07	28.46	46.09	36.52
	26.45	21.89	27.45	29.15
	22.47	20.3	33.13	17.32
	26.23	24.8	46.06	38.6
	36.99	27.1	22.6	31.2
	32.59	21.04	30.9	19.2
	30.3	31.85	20.5	30.1
	29.9	41.74	24.4	26.5
	28.3		14.9	43.75
	20.42		27.2	49.54
	40.02		46.47	
	25.91		29.79	
	36.3		42.33	
			46.3	
**IFN-y+ TNF-a+**	2.17	7.45	1.91	2.3
	1.97	3.16	0.39	2.72
	6.35	9.59	1.05	1.75
	3.67	11.8	1.93	0.62
	3.23	19.6	1.06	26.5
	7.39	20.9	14.1	23
	3.79	3.54	22.5	12.3
	26.7	4.45	12.5	18.4
	18.2	9.34	20.2	19.1
	22.4		10.2	4.35
	1.92		19.3	2.84
	6.62		8.27	
	2.31		9.19	
	5.9		4.73	
			4.4	

Fig EV1 I - Tumor CD4 cells freq

	CD4cre Ptpn6/11$^{wt/wt}$ Iso	CD4cre Ptpn6/11$^{wt/wt}$ aPD1	CD4cre Ptpn6/11$^{fl/fl}$ Iso	CD4cre Ptpn6/11$^{fl/fl}$ aPD1
**CD4+**	13.5	40.1	10.6	12.3
	14.4	23.8	11.6	8.38
	25.3	23.1	6.04	8.39
	16.2	13.4	13.4	10.2
	20.6	19.3	19.9	16
	23.1	11.8	19.6	14.4
	23.5	23	14.3	12.5
	18.8	23.4	17.7	23.3
	15.2	27	22	35.4
	14.4	15.5	16.6	22.3
	27.9		21.6	
	24.4		16.4	
	16		24.8	
	23.8		22.5	
