# Supplementary material for: Concomitant deletion of Ptpn6 and Ptpn11 in T cells fails to improve anticancer responses
Source: EMBO Rep. 2022 Oct 4;23(11):e55399. doi: 10.15252/embr.202255399 (PMC9638855; doi:10.15252/embr.202255399)
Supplement: Supplementary file 3 — Source Data for Expanded View [file EMBR-23-e55399-s008.zip › Fig EV2.pdf]

Fig EV2 A - Spleen counts

	$Ptpn6/11^{fl/fl}$	GzmBcre $Ptpn6/11^{fl/fl}$
Cellularity	67944800	59011400
	61931600	53211000
	51547400	68436400
	62775400	47353000
	63087000	55220000
	61526000	62797200
	52445000	
	$Ptpn6/11^{fl/fl}$	GzmBcre $Ptpn6/11^{fl/fl}$
CD4 numbers	5930200	3649800
	4531400	4052000
	2231800	4859600
	5446600	4216200
	5174200	4218600
	3001400	4075800
	2491000	
	$Ptpn6/11^{fl/fl}$	GzmBcre $Ptpn6/11^{fl/fl}$
CD8 numbers	2584800	1991600
	2058400	2386000
	966800	1711400
	2662000	2091600
	2385400	2514400
	1637200	1953800
	1501200	

Fig EV2 B - LN counts

	$Ptpn6/11^{fl/fl}$	GzmBcre $Ptpn6/11^{fl/fl}$
Cellularity	792624	716328
	657184	636488
	614748	434704
	836000	432416
	689982	994000
	746290	754504
	647206	
	$Ptpn6/11^{fl/fl}$	GzmBcre $Ptpn6/11^{fl/fl}$
CD4 numbers	72586	78370
	63944	53718
	57382	51028
	130464	33916
	49822	125652
	90038	97870
	90382	
	$Ptpn6/11^{fl/fl}$	GzmBcre $Ptpn6/11^{fl/fl}$
CD8 numbers	68002	83962
	42568	57146
	64056	46912
	108770	31000
	46824	106062
	85362	79982
	80982	

Fig EV2 C - Spleen CD44 exp

	$Ptpn6/11^{fl/fl}$	GzmBcre $Ptpn6/11^{fl/fl}$
CD4+ CD44 exp	19.19	25.05
	26.11	23.14
	29.16	23.01
	25.96	23.95
	$Ptpn6/11^{fl/fl}$	GzmBcre $Ptpn6/11^{fl/fl}$
CD8+ CD44 exp	19.03	23.58
	25.41	18.96
	29.1	16.99
	27.66	19.4

Fig EV2 D - LN CD44 exp

	$Ptpn6/11^{fl/fl}$	GzmBcre $Ptpn6/11^{fl/fl}$
CD4+ CD44 exp	11.03	10.83
	12.64	11.92
	15.64	12.05
	9.66	12.69
	$Ptpn6/11^{fl/fl}$	GzmBcre $Ptpn6/11^{fl/fl}$
CD8+ CD44 exp	16.66	20.46
	16.31	17.76
	19.48	15.13
	15.52	16.12
