# Supplementary material for: Concomitant deletion of Ptpn6 and Ptpn11 in T cells fails to improve anticancer responses
Source: EMBO Rep. 2022 Oct 4;23(11):e55399. doi: 10.15252/embr.202255399 (PMC9638855; doi:10.15252/embr.202255399)
Supplement: Supplementary file 3 — Source Data for Expanded View [file EMBR-23-e55399-s008.zip › Fig EV3.pdf]

Fig EV3 A - Day 2

	$Ptpn6/11^{fl/fl}$	GzmBcre $Ptpn6/11^{fl/fl}$
GITR MFI	169983	195937
	147347	191158
	170015	190862
	$Ptpn6/11^{fl/fl}$	GzmBcre $Ptpn6/11^{fl/fl}$
CD71 MFI	297079	282660
	333797	326633
	389937	310404

Fig EV3 B - Day 5

	$Ptpn6/11^{fl/fl}$	GzmBcre $Ptpn6/11^{fl/fl}$
GITR MFI	49085	48493
	45305	48940
	50455	46378
	$Ptpn6/11^{fl/fl}$	GzmBcre $Ptpn6/11^{fl/fl}$
CD71 MFI	36540	39147
	37330	38392
	32958	38595
	$Ptpn6/11^{fl/fl}$	GzmBcre $Ptpn6/11^{fl/fl}$
CD69 MFI	3449	3061
	3444	2834
	2940	3242
	$Ptpn6/11^{fl/fl}$	GzmBcre $Ptpn6/11^{fl/fl}$
CD44 MFI	25111	25832
	29322	28608
	26301	27970

Fig EV3 C - G1 phase

	$Ptpn6/11^{fl/fl}$	GzmBcre $Ptpn6/11^{fl/fl}$
Day 4	45	45
	44.1	43.2
	47.3	42
Day 5	42.4	40.5
	43.4	42.9
	44.2	43.9
Day 6	54	53.6
	52.4	54.4
	54.9	54.6

Fig EV3 C - S phase

	$Ptpn6/11^{fl/fl}$	GzmBcre $Ptpn6/11^{fl/fl}$
Day 4	43.2	45
	43.8	43.5
	40.8	43.7
Day 5	46.8	48.4
	45.3	43.9
	43.6	44.2
Day 6	35.6	36.6
	39.4	34.8
	36.7	35.1

Fig EV3 C - G2 phase

	$Ptpn6/11^{fl/fl}$	GzmBcre $Ptpn6/11^{fl/fl}$
Day 4	5.41	2.85
	6.29	5
	5.59	3.99
Day 5	4.75	2.5
	6.56	5.39
	6.25	4.55
Day 6	6.06	4.67
	3.94	5.18
	4.43	5.06
