# Supplementary material for: Concomitant deletion of Ptpn6 and Ptpn11 in T cells fails to improve anticancer responses
Source: EMBO Rep. 2022 Oct 4;23(11):e55399. doi: 10.15252/embr.202255399 (PMC9638855; doi:10.15252/embr.202255399)
Supplement: Supplementary file 4 — PDF+ [file EMBR-23-e55399-s005.pdf]

Report

SOURCE DATA | TRANSPARENT PROCESS | OPEN ACCESS

EMBO reports

Concomitant deletion of Ptpn6 and Ptpn11 in T cells fails to improve anticancer responses

Pedro M O Ventura[1,†], Milica Gakovic[2,†], Berenice A Fischer[1], Laura Spinelli[2], Giorgia Rota[3], Shalini Pathak[2], Hanif J Khameneh[1], Alessandro Zenobi[1], Sarah Thomson[4], Walter Birchmeier[5], Doreen A Cantrell[2,*,‡] & Greta Guarda[1,**,‡]

Abstract

Anticancer T cells acquire a dysfunctional state characterized by poor effector function and expression of inhibitory receptors, such as PD-1. Blockade of PD-1 leads to T cell reinvigoration and is increasingly applied as an effective anticancer treatment. Recent work challenged the commonly held view that the phosphatase PTPN11 (known as SHP-2) is essential for PD-1 signaling in T cells, suggesting functional redundancy with the homologous phosphatase PTPN6 (SHP-1). Therefore, we investigated the effect of concomitant Ptpn6 and Ptpn11 deletion in T cells on their ability to mount antitumour responses. *In vivo* data show that neither sustained nor acute Ptpn6/11 deletion improves T cell-mediated tumor control. Sustained loss of Ptpn6/11 also impairs the therapeutic effects of anti-PD1 treatment. *In vitro* results show that Ptpn6/11-deleted CD8+ T cells exhibit impaired expansion due to a survival defect and proteomics analyses reveal substantial alterations, including in apoptosis-related pathways. These data indicate that concomitant ablation of Ptpn6/11 in polyclonal T cells fails to improve their anticancer properties, implying that caution shall be taken when considering their inhibition for immunotherapeutic approaches.

Keywords PD-1 checkpoint blockade; Ptpn11; Ptpn6; T cell exhaustion
Subject Categories Cancer; Immunology; Signal Transduction
DOI 10.15252/embr.202255399 | Received 16 May 2022 | Revised 5 September 2022 | Accepted 8 September 2022 | Published online 4 October 2022
EMBO Reports (2022) 23: e55399

Introduction

CD8+ effector T cells are essential cytotoxic lymphocytes that kill pathogen-infected cells and tumors. Their expansion and differentiation is controlled by signal transduction pathways initiated by antigenic, co-stimulatory, and cytokine signals and modulated by inhibitory immune checkpoint receptors (Wherry & Kurachi, 2015). These inhibitory receptors are expressed at high levels in chronically stimulated T cells and function to dampen T cell activation to prevent autoimmune pathology during chronic viral infection. However, they also limit antitumour T cell responses. Hence, the blockade of inhibitory receptors such as programmed cell death protein 1 (PD-1) is emerging as the key to successful cancer immunotherapy (Topalian, 2017). This has prompted a focus on the intracellular signaling modules that mediate inhibition of T cell activation. In this context, cytosolic tyrosine kinases such as lymphocyte-specific protein tyrosine kinase (LCK) and Zeta-chain-associated protein kinase 70 (ZAP-70) are the initiators of T cell activation, whereas cytosolic tyrosine phosphatases are proposed to mediate the actions of inhibitory receptors (Gaud *et al*, 2018; Niogret *et al*, 2019a). Two homologous molecules thought to mediate negative feedback control of activated T cells are the cytoplasmic Src homology 2 (SH2) domain–containing phosphatases-1 (SHP-1), also known as and hereafter called PTPN6, and SHP-2, hereafter called PTPN11 (Poole & Jones, 2005; Yuan *et al*, 2020). These phosphatases are recruited into signalosomes via their SH2 domains, which bind to immunoreceptor tyrosine-based inhibitory motif (ITIM) and immunoreceptor tyrosine-based switch motif (ITSM) found in the cytoplasmic tail of inhibitory receptors such as PD-1 (Chemnitz *et al*, 2004; Sheppard *et al*, 2004; Yokosuka *et al*, 2012; Celis-Gutierrez *et al*, 2019; Marasco *et al*, 2020). PTPN6 modulates antigen receptor responses by targeting LCK and ZAP70 as well as adaptors in the pathway (Gaud *et al*, 2018). The proposal for PTPN11 is that it suppresses T cell function primarily by inactivating the co-stimulatory CD28 signaling (Hui *et al*, 2017; Kamphorst *et al*, 2017).

How good is the evidence that PTPN6 and PTPN11 mediate the actions of inhibitory receptors and restrict CD8+ T cell effector function? Key observations are that Ptpn6 deletion in transgenic T cells causes enhanced sensitivity to antigen receptor engagement,

1 Institute for Research in Biomedicine, Università della Svizzera Italiana, Bellinzona, Switzerland
2 Cell Signalling and Immunology Division, School of Life Sciences, University of Dundee, Dundee, UK
3 Department of Biochemistry, University of Lausanne, Epalinges, Switzerland
4 Biological Services, University of Dundee, Dundee, UK
5 Max-Delbrueck-Center for Molecular Medicine (MDC) in the Helmholtz Society, Berlin, Germany
*Corresponding author. Tel: +44 1382 385156; E-mail: d.a.cantrell@dundee.ac.uk
**Corresponding author. Tel: +41 58 666 7227; E-mail: greta.guarda@irb.usi.ch
†These authors contributed equally to this work
‡These authors contributed equally to this work as senior authors

© 2022 The Authors. Published under the terms of the CC BY 4.0 license.

promotes the production of short-lived $CD8^+$ T effector cells, and improves the efficacy of adoptive immunotherapy against a mouse model of leukemia (Fowler *et al*, 2010; Stromnes *et al*, 2012). However, another study showed that Ptpn6 knockdown did not promote anti-tumor immunity against B16 melanoma by transgenic T cells, unless checkpoint blockade was administered (Snook *et al*, 2020), raising questions on the conditions in which PTPN6 is relevant for antitumor T cell immunity. Instead, the evidence that PTPN11 suppresses $CD8^+$ T cell immune responses to tumors stems largely from PTPN11 binding to inhibitory receptor complexes (Chemnitz *et al*, 2004; Sheppard *et al*, 2004; Yokosuka *et al*, 2012; Celis-Gutierrez *et al*, 2019; Marasco *et al*, 2020) rather than from evidence of enhanced anticancer T cell responses caused by its deletion. Indeed, the best-known function of PTPN11 is to act as a positive transducer of proliferative and antiapoptotic signals, notably in the context of the Ras/mitogen-activated protein kinase (MAPK) pathway (Dance *et al*, 2008; Niogret *et al*, 2019b). Moreover, T cell-specific Ptpn11 deficiency does not prevent T cell exhaustion, or the therapeutic effects of PD-1 blockade on the control of solid tumors (Rota *et al*, 2018). *In vitro* data suggested that the failure of PTPN11 deletion to improve the efficacy of effector $CD8^+$ T cells is due to the redundancy between PTPN11 and PTPN6 (Celis-Gutierrez *et al*, 2019). This concept has been challenged by recent observations that PD-1 engagement can still suppress the *in vitro* activation of T cells lacking both phosphatases (Xu *et al*, 2020). However, the critical test of whether the combined loss of Ptpn6 and Ptpn11 promotes the ability of $CD8^+$ T cells to clear solid tumors has not been done. Accordingly, the objective of the present study was to assess first the impact of the intrinsic loss of Ptpn6 and then of Ptpn6 and Ptpn11 on T cell-mediated immune responses to tumors.

Results and Discussion

T cell-specific deletion of Ptpn6 and Ptpn11 does not enhance MC38 tumor clearance

To start with, we generated mice with a T cell-specific ablation of Ptpn6 by using the *CD4cre* deleter strain and the efficiency of deletion was confirmed by western blot (Fig 1A and B). In line with previous reports (Johnson *et al*, 2013; Martinez *et al*, 2016), we found that *CD4cre Ptpn6*$^{fl/fl}$ mice exhibited peripheral $CD4^+$ and $CD8^+$ T cells with higher levels of the activation marker CD44 and decreased $CD4^+$ T cells in lymph nodes (Fig EV1A and B). Additionally, splenic regulatory T cell (Treg) numbers were not significantly altered despite a mild increase in frequency (Fig EV1A). We thus tested the ability of *CD4cre Ptpn6*$^{fl/fl}$ mice to control the growth of the MC38 colon adenocarcinoma cells, an immunogenic cancer model that is controlled following blockade of PD-1 (Juneja *et al*, 2017). *CD4cre Ptpn6*$^{fl/fl}$ and control mice were subcutaneously engrafted with MC38 cells and injected either with PD-1 antibody monotherapy or with isotype control antibodies. Our data showed that tumors grew equally in control versus *CD4cre Ptpn6*$^{fl/fl}$ mice (Fig 1C and D). When control mice were treated with PD-1 antibodies, there was a delay in tumor growth. Strikingly, the efficacy of PD-1 monotherapy was reduced in mice where T cells lacked Ptpn6 expression (Fig 1C and D). These results unexpectedly show that deletion of Ptpn6 in T cells does not improve cancer control, rather affecting the response to checkpoint blockade.

To next test if Ptpn6 and Ptpn11 have redundant roles in anticancer T cells, we generated two independent T cell-specific knockout strains (Fig 1A). *CD4cre* mice were crossed onto the *Ptpn6*$^{fl/fl}$ and the *Ptpn11*$^{fl/fl}$ strain, in which exons 3 and 4 are floxed (Grossmann *et al*, 2009), hereafter called *Ptpn11*$^{fl/flWbm}$. *CD4cre* mice were also crossed onto *Ptpn6*$^{fl/fl}$ and the *Ptpn11*$^{fl/fl}$ strain, in which exon 11 is floxed (Zhang *et al*, 2004), referred to as *Ptpn11*$^{fl/flBgn}$. Ptpn6 and Ptpn11 deletion efficiency in T cells from *CD4cre Ptpn6*$^{fl/fl}$*/Ptpn11*$^{fl/flWbm}$ mice was confirmed by western blot (Fig 1B). In line with data from *CD4cre Ptpn6*$^{fl/fl}$ mice (Martinez *et al*, 2016), *CD4cre Ptpn6*$^{fl/fl}$*/Ptpn11*$^{fl/flWbm}$ mice presented reduced output of mature thymocytes (Fig EV1C). Conventional T cell and Treg numbers were not significantly altered in the spleens of *CD4cre Ptpn6*$^{fl/fl}$*/Ptpn11*$^{fl/flWbm}$ mice (Figs 1E and F, and EV1D), although frequency of the latter was increased. As with the *CD4cre Ptpn6*$^{fl/fl}$ mice, both $CD4^+$ and $CD8^+$ T cells exhibited high levels of CD44 (Figs 1E and F, and EV1E). Furthermore, a marked reduction in T cell numbers in lymph nodes isolated from *CD4cre Ptpn6*$^{fl/fl}$*/Ptpn11*$^{fl/flWbm}$ was noticed (Fig EV1E). When inoculated with MC38 cells, neither *CD4cre Ptpn6*$^{fl/fl}$*/Ptpn11*$^{fl/flWbm}$ (Fig 1G and H) nor *CD4cre Ptpn6*$^{fl/fl}$*/Ptpn11*$^{fl/flBgn}$ mice (Fig EV1F) were able to control tumor growth

Figure 1. *CD4cre Ptpn6*$^{fl/fl}$ mice and *CD4cre Ptpn6/11*$^{fl/fl}$ mice do not exhibit superior antitumor immunity.

A The schematic illustrates *CD4cre*-mediated deletion of Ptpn6 or Ptpn6/11 during thymic T cell development; double-negative thymocytes (DN) and double-positive thymocytes (DP) are depicted. Created with Biorender (Biorender.com).

B Total T cells were enriched from spleens of *Ptpn6*$^{fl/fl}$*/Ptpn11*$^{fl/flWbm}$, *CD4cre Ptpn6*$^{fl/fl}$*/Ptpn11*$^{fl/flWbm}$, *CD4cre Ptpn6*$^{fl/fl}$ mice. Expression of Ptpn6 and Ptpn11 was tested by immunoblot in cell lysates, Gapdh was used as loading control.

C, D *CD4cre Ptpn6*$^{fl/fl}$ and control mice were subcutaneously injected with MC38 cells. Tumor growth in individual mice is shown for the indicated genotypes and treatments; number of mice eradicating the tumor is shown within the graphs (C). Survival curves are shown (D).

E, F A representative flow cytometry plot (E), spleen cellularity, $CD4^+$ T cell (gated as $TCR\beta^+$ $CD4^+$) numbers, $CD8^+$ T cell (gated as $TCR\beta^+$ $CD8^+$) numbers, and percentages of $CD44^+$ T cells (F) from the spleens of *CD4cre Ptpn6*$^{fl/fl}$*/Ptpn11*$^{fl/flWbm}$ and control mice are shown.

G, H Tumor growth in individual mice challenged with MC38 and subsequently treated with anti-PD-1 antibody or isotype control is shown for each indicated genotype; number of mice eradicating the tumor is shown within the graphs (G). Survival curves of *CD4cre Ptpn6*$^{fl/fl}$*/Ptpn11*$^{fl/flWbm}$ and control mice is shown (H).

I, J Ten to twelve days following MC38 tumor inoculation and treatment with anti-PD-1 antibody or isotype control, *CD4cre Ptpn6/11*$^{wt/wt}$ and *CD4cre Ptpn6*$^{fl/fl}$*/Ptpn11*$^{fl/flWbm}$ mice were sacrificed, and TILs analyzed. Graphs depict frequencies of $CD8^+$ T cells (gated as $CD45^+$ $TCR\beta^+$ $CD8^+$) expressing IFN-γ and TNFα upon re-stimulation (I) and percentages of $CD8^+$ T cells (J).

Data information: Results depict n = 8–10 mice/group (C, D) and n = 8–11 mice/group (G, H); statistical significance was calculated by log-rank (Mantel-Cox) test (D, H). Results depict mean ± SEM of n = 10–12 mice/group (F) and of n = 9–14 mice/group (I, J). Student's t-test (unpaired, two-tailed) was used to compare differences between experimental groups (F, I, J). $*P \le 0.05$, $**P \le 0.01$, $***P \le 0.001$, $****P \le 0.0001$.
Source data are available online for this figure.

© 2022 The Authors

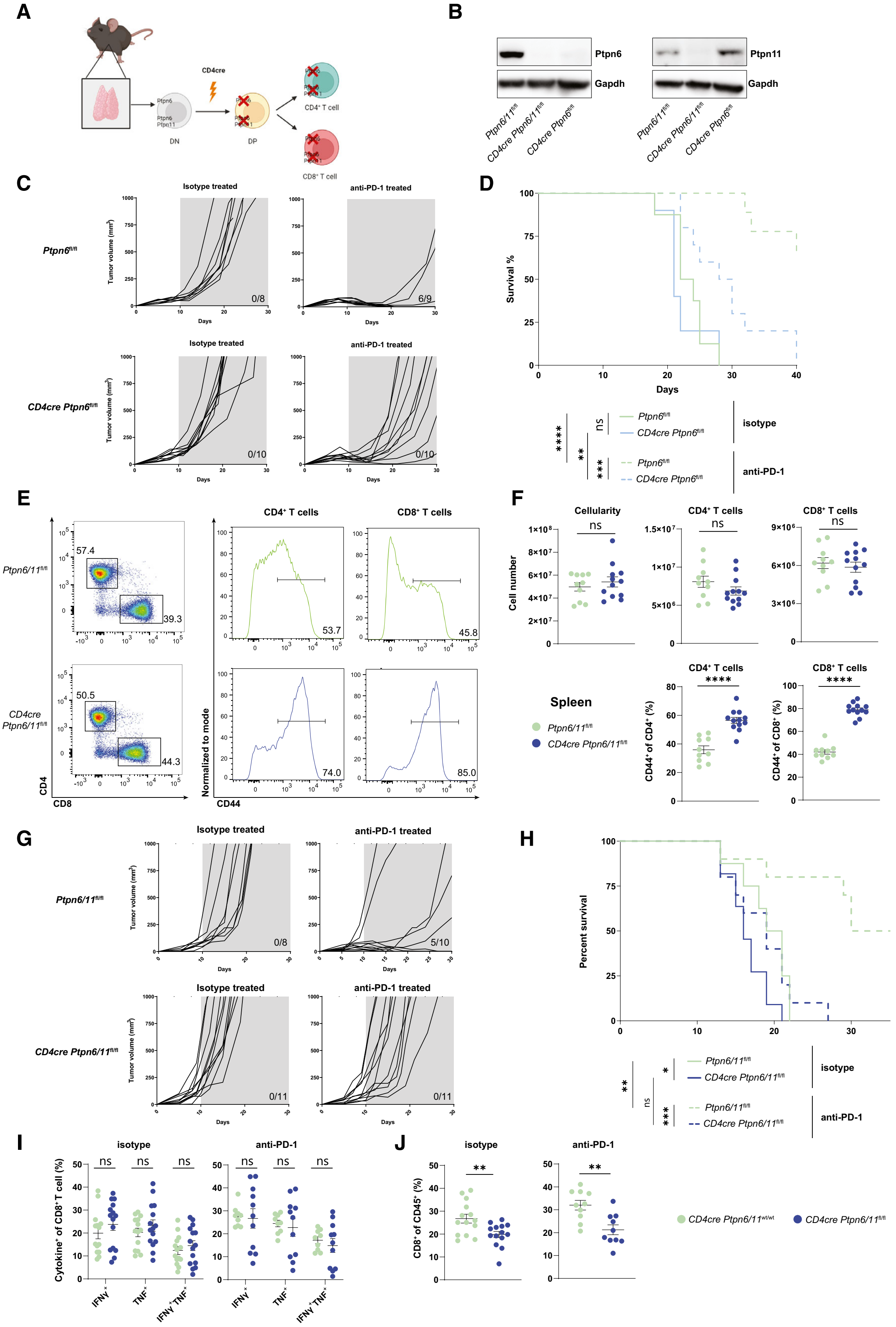

A
CD4cre
DN
DP
CD4+ T cell
CD8+ T cell
B
Ptpn6
Gapdh
Ptpn11
Gapdh
Ptpn6/11fl/fl
CD4cre Ptpn6/11fl/fl
CD4cre Ptpn6fl/fl
C
Isotype treated
anti-PD-1 treated
Ptpn6fl/fl
CD4cre Ptpn6fl/fl
Tumor volume (mm3)
Days
0/8
6/9
0/10
0/10
D
Survival %
Days
Ptpn6fl/fl
CD4cre Ptpn6fl/fl
isotype
anti-PD-1
E
Ptpn6/11fl/fl
CD4cre Ptpn6/11fl/fl
57.4
39.3
50.5
44.3
CD4
CD8
CD4+ T cells
CD8+ T cells
Normalized to mode
CD44
53.7
45.8
74.0
85.0
F
Cellularity
CD4+ T cells
CD8+ T cells
Cell number
ns
Spleen
CD44+ of CD4+ (%)
CD44+ of CD8+ (%)
****
G
Isotype treated
anti-PD-1 treated
Ptpn6/11fl/fl
CD4cre Ptpn6/11fl/fl
Tumor volume (mm3)
Days
0/8
5/10
0/11
0/11
H
Percent survival
isotype
anti-PD-1
I
isotype
anti-PD-1
Cytokine+ of CD8+T cell (%)
IFNγ+
TNF+
IFNγ+TNF+
ns
J
CD8+ of CD45+ (%)
**
CD4cre Ptpn6/11wt/wt
CD4cre Ptpn6/11fl/fl

Figure 1.

better than control mice, rather showing a worsened response. Moreover, control mice showed improved antitumoral responses following PD-1 antibody therapy, while *CD4cre Ptpn6*$^{fl/fl}$/*Ptpn11*$^{fl/flWbm}$ mice did not (Fig 1G and H), despite similar percentage of PD-1$^+$ CD8$^+$ tumor infiltrating lymphocytes (TILs) in both genotypes (Fig EV1G). To explore why Ptpn6 and Ptpn11 ablation in T cells caused reduced antitumoral immunity, we compared the TILs 10–12 days following tumor engraftment in either control or *CD4cre Ptpn6*$^{fl/fl}$/*Ptpn11*$^{fl/flWbm}$ mice. The data showed that while the capacity to produce effector cytokines was not significantly altered in both CD8$^+$ and CD4$^+$ T cells (Figs 1I and EV1H), the frequency of tumor infiltrating CD8$^+$ – but not CD4$^+$ – T cells was reduced in *CD4cre Ptpn6*$^{fl/fl}$/*Ptpn11*$^{fl/flWbm}$ mice compared to control mice (Figs 1J and EV1I). Taken together, these results indicate that Ptnp6/11 null T cells fail to show enhanced anti-tumor effectiveness *in vivo*.

Deletion of Ptpn6 and Ptpn11 in peripheral cytotoxic T cells fails to promote antitumoral immunity

One possibility we considered was that the loss of these two phosphatases in T cells during important steps prior to the cancer challenge, directly or indirectly affected CD8$^+$ T cell functionality. In particular, we wondered if quantitative or qualitative changes in the T cell compartment observed in *CD4cre Ptpn6/11*$^{fl/fl}$ mice might have masked any reinvigorating effect of Ptpn6/11 deletion on tumor-infiltrating T cells. We therefore backcrossed *Ptpn6*$^{fl/fl}$/*Ptpn11*$^{fl/flBgn}$ mice to mice carrying the Cre recombinase under the promoter of *Granzyme B* (*Gzmb*) (Jacob & Baltimore, 1999) (Fig 2A), which is activated as T cells differentiate to cytotoxic cells such as in the context of cancer (Nishida *et al*, 2021). As expected, analysis of steady-state *Gzmbcre Ptpn6*$^{fl/fl}$/*Ptpn11*$^{fl/flBgn}$ (thereafter called *Gzmbcre Ptpn6/11*$^{fl/fl}$) mice did not reveal major alterations (Fig EV2A–D), whereas *in vitro* cultured cytotoxic T lymphocytes (CTLs) showed nearly complete deletion of the two phosphatases (Fig 2B). To determine whether *Gzmbcre* targets the CD8$^+$ T cells infiltrating MC38 tumors, we crossed the *Gzmbcre* mice with reporter mice expressing yellow fluorescent protein (YFP) upon Cre-recombinase activation (Srinivas *et al*, 2001). Half of the tumor-infiltrating CD8$^+$ T cells in these mice expressed detectable YFP and were largely PD1$^+$ (Fig 2C and D), indicating that the *Gzmbcre* system is targeting the expected Gzmb/PD1-positive population within the tumor (Juneja *et al*, 2017). We thus engrafted MC38 tumors in *Gzmbcre Ptpn6/11*$^{fl/fl}$ mice. These mice failed to exhibit improved survival as compared with their control counterparts (Fig 2E), and the time from detection of palpable tumors to experimental endpoint was significantly shorter in *Gzmbcre Ptpn6/11*$^{fl/fl}$ mice compared with *Ptpn6*$^{fl/fl}$ *Ptpn11*$^{fl/flBgn}$ animals (Figs 2F and EV2E). While our data from *CD4cre Ptpn6/11*$^{fl/fl}$ mice might reveal effects contributed by all T cells, including conventional and regulatory CD4$^+$ T cells, from the results obtained with *Gzmbcre Ptpn6/11*$^{fl/fl}$ mice, we can infer that anticancer responses are also not reinvigorated by the acute deletion of Ptpn6 and Ptpn11 in cytotoxic T cells.

Ptpn6 and Ptpn11 are required for proteome homeostasis and survival of CD8$^+$ T cells

Given the failure to improve antitumoral immunity upon deletion of Ptpn6 and Ptpn11 in developing or effector T cells and their reduced number in the tumor, we wondered if the absence of these phosphatases was undermining T cell expansion. We thus used *in vitro* models where naïve CD8$^+$ T cells from *Gzmbcre Ptpn6/11*$^{fl/fl}$ mice were activated with CD3 and CD28 antibodies in the presence of IL-2 and IL-12 and then differentiated into effector cells in the presence of IL-2. The initial activation response of *Gzmbcre Ptpn6/11*$^{fl/fl}$ mice-derived T cells did not present major differences as compared with control T cells in terms of cell size, granularity, expression of the IL-2 receptor subunit CD25, the inhibitory receptors PD-1 and Glucocorticoid-induced TNFR-related protein (GITR), and the transferrin receptor CD71 (Figs 3A and EV3A). After a further 3 days of growth in IL-2, *Gzmbcre Ptpn6/11*$^{fl/fl}$ mice-derived T cells expressed higher cell surface levels of CD25 and PD-1 (Fig 3B), but similar levels of GITR, CD71, and the activation markers CD44 and CD69 (Fig EV3B). However, a striking difference was that Ptpn6/11-deleted T cells showed reduced expansion compared with control cells (Fig 3C).

To understand the phenotype of the Ptpn6/11 null CD8$^+$ T cells further, we used quantitative high-resolution mass spectrometry to resolve their proteome. We identified over 6,500 proteins, estimated protein copy numbers per cell and protein abundance relying on the "proteomic ruler" method, which uses the histone mass spectrometry signal as an internal standard. Of the proteins identified, 313 were downregulated in *Gzmbcre Ptpn6/11*$^{fl/fl}$ T cells, with a further 157 detected only in control T cells; 25 proteins were upregulated in *Gzmbcre Ptpn6/11*$^{fl/fl}$ T cells, with a further 40 proteins detected only in *Gzmbcre Ptpn6/11*$^{fl/fl}$ T cells (Fig 3D; full list of proteins identified in Dataset EV1). Pathway enrichment analysis on the proteins absent or downregulated in Ptpn6/11 null T cells not surprisingly revealed alterations in phosphorylation, but also in transcription, protein transport, cell cycle, as well as apoptosis (Fig 3D and E). We therefore analyzed in detail Ptpn6/11-deleted T cells throughout the *in vitro* culture and found that their decreased expansion was not associated with cell cycle arrest but with increased frequency of cells showing evidence for DNA degradation (a sub G1 peak) and cell death (Figs 3F and G, and EV3C). Accordingly, proteome analysis of Ptpn6/11 null CD8$^+$ T cells demonstrated a substantial decrease in tumor necrosis factor (TNF) receptor superfamily member 1b (Tnfrsf1b, also known as Tnfr2), which favors survival of early effector CD8$^+$ T cells (Kim & Teh, 2004; Calzascia *et al*, 2007), and in myeloid cell leukemia sequence 1 (Mcl-1), an anti-apoptotic protein crucial for the survival of T cells at multiple stages of development (Dzhagalov *et al*, 2008; Carrington *et al*, 2017) (Fig 3H). Together, these data indicate that lack of Ptpn6 and Ptpn11 impairs T cell survival. A reduced survival could explain the decreased tumor infiltration and the lack of improved anticancer immunity observed *in vivo*.

We have addressed here the question of the redundant role of Ptpn6 and Ptpn11 in anti-tumoral T cell responses. We find that deletion of both phosphatases is detrimental for CD8$^+$ T cells, leading to considerable alterations in their proteome, including reduced levels of pro-survival molecules. These cells exhibit increased cell death and consequently reduced expansion *in vitro*. In agreement, tumor infiltration and control of tumor growth are affected in a model of sustained Ptpn6/11 deficiency in T cells, in the presence or absence of anti-PD-1 treatment. Although it is likely that multiple mechanisms – including increased activation induced cell death or terminal differentiation – contribute to their defective antitumoral response, our *in vitro* findings

 © 2022 The Authors

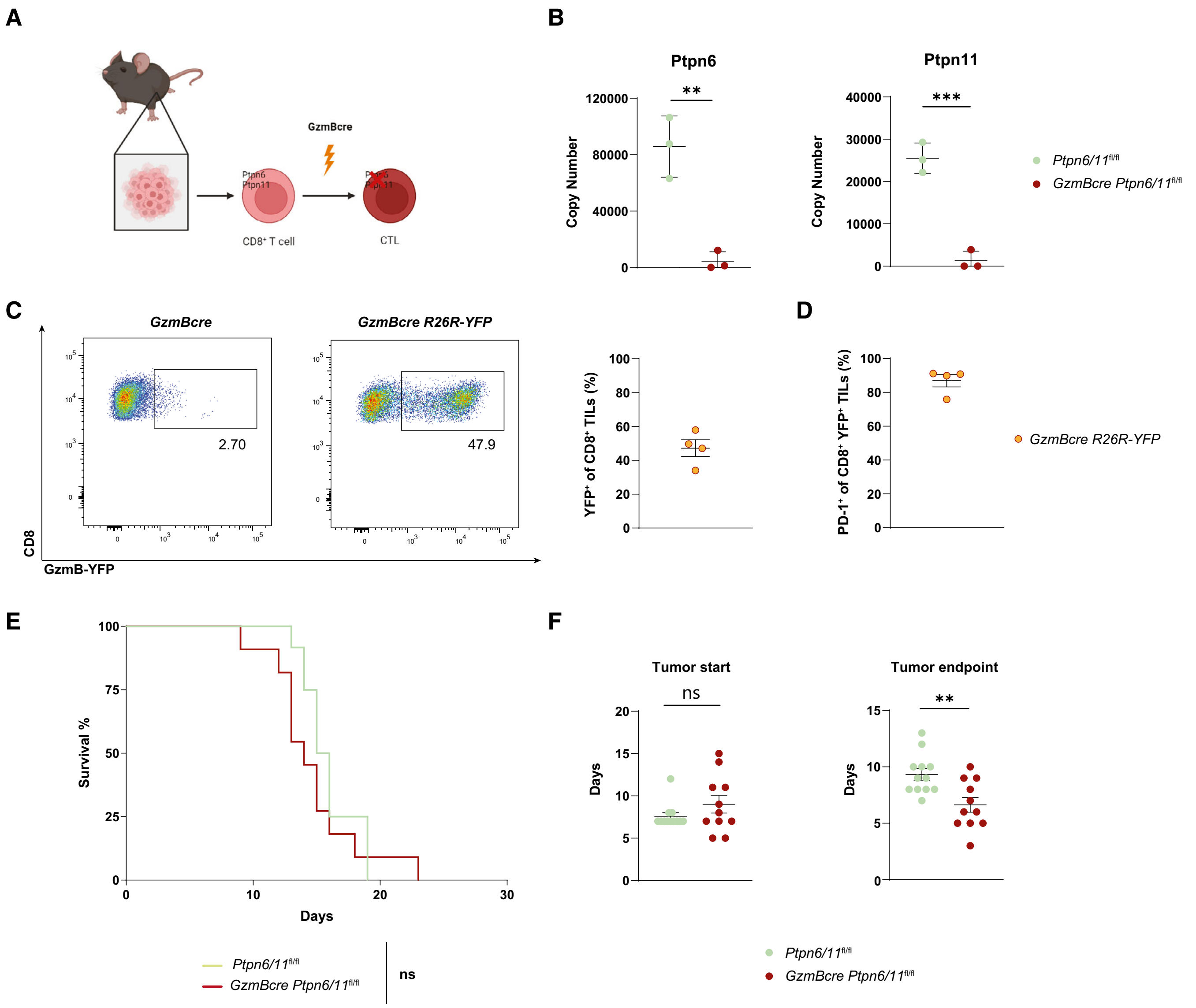

Figure 2. Deletion of Ptpn6/11 in effector cells does not lead to enhanced antitumour response.

A The schematic illustrates *GzmBcre*-mediated deletion of Ptpn6/11 following cancer challenge; $CD8^+$ T cells and cytotoxic T lymphocytes (CTLs) are depicted. Created with Biorender (Biorender.com).

B Ptpn6 and Ptpn11 copy number, as estimated through high resolution mass spectrometry in *GzmBcre Ptpn6/11*$^{fl/fl}$ and control CTLs.

C, D *GzmBcre R26R-YFP* reporter mice were injected subcutaneously with MC38 cells and analyzed after 11 days. Graphs show the percentage of YFP^+ cells among $CD45^+$ $CD8^+$ TILs (C) and the percentage of PD-1^+ cells among $CD45^+$ $CD8^+$ YFP^+ TILs (D). Results depict n = 4 mice (C, D).

E, F *GzmBcre Ptpn6/11*$^{fl/fl}$ and control *Ptpn6/11*$^{fl/fl}$ mice were subcutaneously injected with MC38 cells. Survival curves and statistical comparisons between different groups are shown (E). The tumor start depicts the time (days) following tumor engraftment after which the tumor is palpable, while tumor endpoint depicts the time (days) from tumor start until the reaching the maximal allowed tumor size (F).

Data information: Results depict mean ± SD of n = 3 biological replicates (B), mean ± SEM of n = 4 biological replicates (C, D), mean ± SEM (F) of n = 11–12 mice/group (E, F); statistical significance was calculated by log-rank (Mantel-Cox) test (E), and Student's t-test (unpaired, two-tailed) was used to compare differences between experimental groups (B, F). $^{**}P \leq 0.01$, $^{***}P \leq 0.001$.

Source data are available online for this figure.

that Ptpn6/11-deleted T cells exhibit increased cell death provide a plausible explanation for the observations *in vivo*. Importantly, recent *in vitro* data by Xu and colleagues showed that PD-1-mediated inhibition still occurs in T cells lacking both phosphatases (Xu *et al*, 2020). Together, these results indicate that the tumor-suppressing effect of anti-PD-1 antibodies is unlikely to be mediated exclusively by Ptpn6 and Ptpn11 in T cells, implying the involvement of additional molecules. Moreover, a recent report demonstrated that myeloid-specific PD-1 deletion decreased tumor growth more efficiently than T cell-specific PD-1 deletion (Strauss *et al*, 2020), suggesting that the effects of PD-1 blockade are not solely mediated by T cells but also by other cells present in the tumor microenvironment.

© 2022 The Authors

Similar to the deletion of Ptpn6/11, deletion of Ptpn6 in our system also fails to improve antitumor responses and impairs the therapeutic effect of PD-1 blockade, indicating that Ptpn6 is important for adequate T cell responses and/or for response to anti-PD-1. Of note, *CD4cre Ptpn11*$^{fl/fl}$ mice showed normal responses to PD-1 blockade (Rota *et al*, 2018). Therefore, while the phenotype of mice with Ptpn6/11-deleted T cells suggests some redundancy in the activities of these phosphatases (Celis-Gutierrez *et al*, 2019), differences between *CD4cre Ptpn6*$^{fl/fl}$ and *CD4cre Ptpn11*$^{fl/fl}$ mice also underline the distinct functions of these phosphatases.

Studies by others on Ptpn6 in anticancer T cell immunity delivered contrasting results, showing that its deletion improved

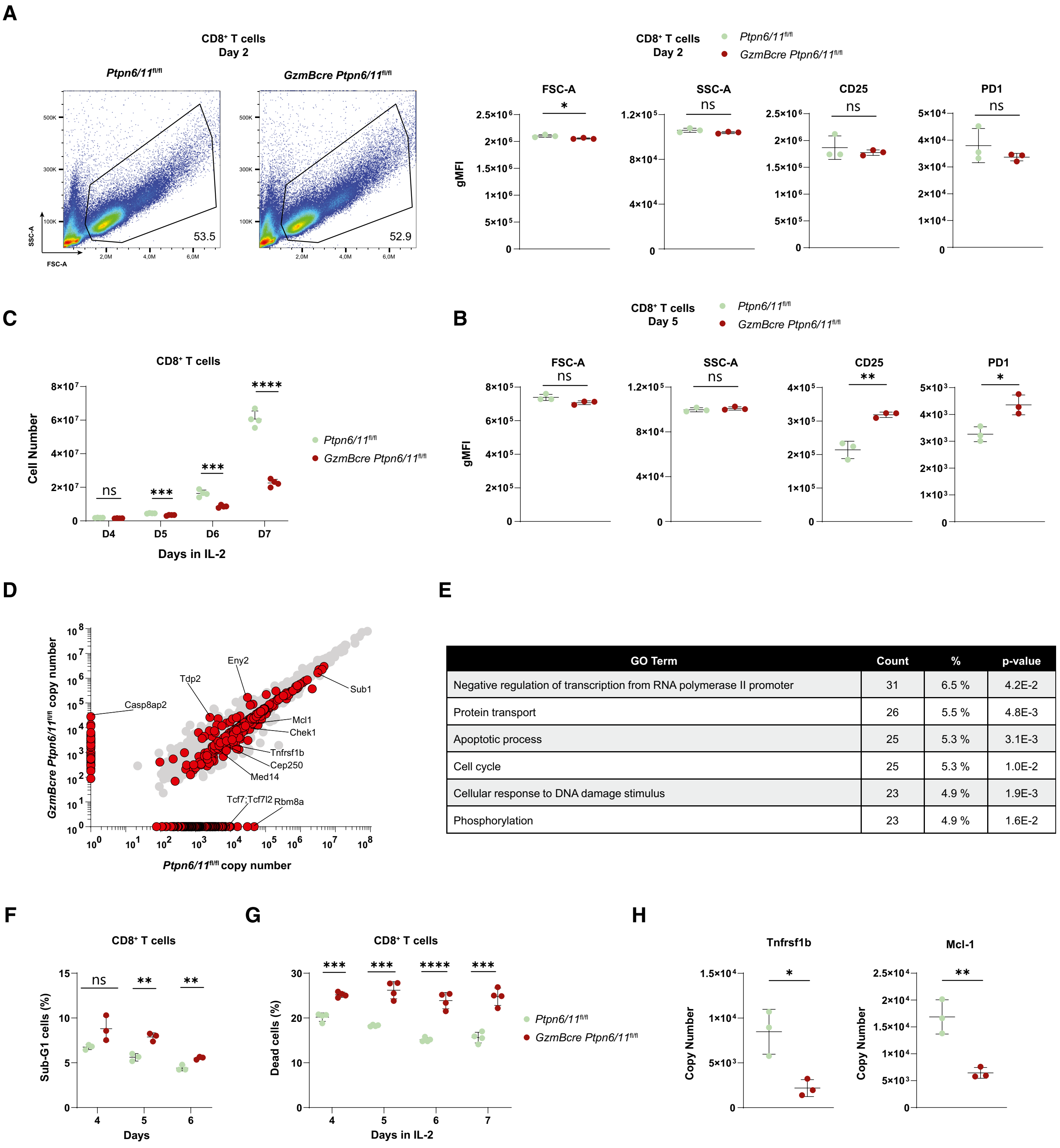

GO Term	Count	%	p-value
Negative regulation of transcription from RNA polymerase II promoter	31	6.5 %	4.2E-2
Protein transport	26	5.5 %	4.8E-3
Apoptotic process	25	5.3 %	3.1E-3
Cell cycle	25	5.3 %	1.0E-2
Cellular response to DNA damage stimulus	23	4.9 %	1.9E-3
Phosphorylation	23	4.9 %	1.6E-2

Figure 3.

© 2022 The Authors

Figure 3. Deletion of Ptpn6 and Ptpn11 impacts on CD8+ T cell proteome and cell survival.

CTLs were generated from splenocytes of *GzmBcre Ptpn6/11fl/fl* and *Ptpn6/11fl/fl* mice by anti-CD3, anti-CD28, and IL-12 stimulation and maintained in IL-2.

A, B CD8+ T cells (gated DAPI− cells) were analyzed by flow cytometry for size, granularity, and surface expression of CD25 and PD-1 after 2 days (A) and after 5 days (B); a quantification of these parameters is shown in the graphs (A, B) and a representative flow cytometry plot is shown for FSC and SSC (A).

C Numbers of CTLs (gated on CD8+ T cells) from *Ptpn6/11fl/fl* or *GzmBcre Ptpn6/11fl/fl* mice were counted daily by flow cytometry with the addition of DAPI to monitor dead cells.

D Quantitative high-resolution mass spectrometry was used to resolve the proteome of *Ptpn6/11fl/fl* and *GzmBcre Ptpn6/11fl/fl* CTLs (day 7). The plot depicts protein copy number in control and *GzmBcre Ptpn6/11fl/fl* cells. Significantly altered proteins are indicated in red; selected examples are annotated. Results are based on $n = 3$ biological replicates and two-tailed, unequal-variance t-test on log10 transformed copy number per cell values was used to compare differences between experimental groups.

E GO Term analysis shows the enriched pathways with at least 23 proteins and a P-value < 0.05, % indicate the % altered genes on the total genes in a given pathway.

F Graph shows the percentage of sub-G1 cells as measured by flow cytometry-based cell cycle analysis of *Ptpn6/11fl/fl* and *GzmBcre Ptpn6/11fl/fl* CTLs (gated on CD8+ T cells) at the indicated days.

G Graph illustrates the percentage of DAPI+ dead cells of *Ptpn6/11fl/fl* and *GzmBcre Ptpn6/11fl/fl* CTLs (gated on CD8+ T cells) at the indicated days as measured by flow cytometry.

H Protein copy number of Tnfrsf1b and Mcl-1 in *GzmBcre Ptpn6/11fl/fl* and control CTLs, as estimated through high resolution mass spectrometry.

Data information: Results depict mean ± SD of $n = 3$ (A, B, F, H) or 4 (C, G) biological replicates and Student's t-test (unpaired, two-tailed) was used to compare differences between experimental groups (A–C, F–H). *$P \le 0.05$, **$P \le 0.01$, ***$P \le 0.001$, ****$P \le 0.0001$.
Source data are available online for this figure.

anticancer T cell responses or did so for low affinity TCR transgenic cells upon checkpoint blockade (Stromnes *et al*, 2012; Snook *et al*, 2020). Notably, in these studies, Ptpn6 was targeted in TCR-transgenic T cells, which were adoptively transferred (Stromnes *et al*, 2012; Snook *et al*, 2020). This suggests that the antigen receptor represents an important determinant of the effects of Ptpn6 deletion and that transfer of CD8+ T cells reduces potentially confounding effects by other T cell subsets affected by this deletion (Iype *et al*, 2010). Accordingly, PTPN6 deletion in genetically engineered T cells might benefit adoptive immunotherapy approaches where predefined antigen receptors are utilized, such as chimeric antigen receptor-based therapies (Hebeisen *et al*, 2013; Rafiq *et al*, 2020).

However, in the current work, we explore the impact of Ptpn6 and Ptpn6/11 loss on the polyclonal T cell population, enabling us to encompass the breadth of their functions in endogenous T cells (Zhang *et al*, 1999; Iype *et al*, 2010; Martinez *et al*, 2016). This gives us insights into the potential effects that small molecule inhibitors blocking both phosphatases might have on endogenous antitumour T cell responses. With PTPN11 inhibitors showing promise against selected cancer types and the ongoing efforts aimed at targeting PTPN6 (Mullard, 2018; Varone *et al*, 2020; Yuan *et al*, 2020), our data suggest that combined blockade of both phosphatases shall be carefully considered in relation to the antitumoral effects of the endogenous T cell population. These results further highlights the need of elucidating cellular and molecular mechanisms behind PD-1 function, an important area of research for the development of small molecule inhibitor-based approaches targeting this inhibitory pathway.

Materials and Methods

Mice

Ptpn6fl/fl mice were obtained from the Jackson Laboratory (JAX stock #008336) and were on a C57BL/6 background (Viant *et al*, 2014). Ptpn11fl/flWbm mice (Grossmann *et al*, 2009) were on a mixed C57BL/6 background and crossed at least six additional times on C57BL/6. *CD4cre* deleter strain, from Jackson Laboratory, were on a C57BL/6 background and were crossed onto Ptpn6fl/fl, and Ptpn6fl/fl/Ptpn11fl/flWbm mice. Male and female 6- to 12-week-old mice were used in different experiments with appropriate sex and age-matched controls. Mouse studies were approved by the Veterinary Office regulations of the State of Ticino, Switzerland, and all methods were performed in accordance with the Swiss guidelines and regulations. Ptpn11fl/flBgn mice were on a C57BL/6J background (Zhang *et al*, 2004) and were crossed to Ptpn6fl/fl, *CD4cre*, and *Gzmbcre* mice (Jacob & Baltimore, 1999). Male and female 6- to 12-week-old mice were used in different experiments with appropriate sex- and age-matched controls. Mice were bred and maintained at the University of Dundee in compliance with UK Home Office Animals (Scientific Procedures) Act 1986 guidelines.

Engrafted tumors

The colon adenocarcinoma cell line MC38 was grown in standard growing conditions at 37°C in 5% CO^2, in a monolayer with DMEM supplemented with 10% fetal calf serum (FCS), 100 U/ml of penicillin, and 100 mg/ml of streptomycin (Gibco). With respect to *CD4cre Ptpn6fl/fl/Ptpn11fl/flWbm*, *CD4cre Ptpn6fl/fl*, and relative control mice, tumor cells were resuspended in PBS, and 4.5×10^5 MC38 cells were injected subcutaneously (s.c.) in the flank. Tumor volume was calculated using the formula $V = (L \times l^2)/2$, where L is the widest diameter and l is the smallest diameter. Animals were sacrificed when tumor volume reached 1,000 mm^3. Five days post-tumor engraftment, when tumors were palpable, mice were treated intraperitoneally with a-PD-1 (RMP1-14, BioXcell) or isotype control (2A3, BioXcell). The treatment was repeated two times at an interval of 3–4 days for a total of three injections of 200 µg/mouse. With respect with *CD4cre Ptpn6fl/fl/Ptpn11fl/flBgn*, *Gzmbcre Ptpn6fl/fl/Ptpn11fl/flBgn*, and relative control mice, tumor cells were resuspended in PBS, and 2×10^5 MC38 cells were injected s.c. in the flank. Tumor volume was calculated using the formula $V = (L \times l^2)/2$. Animals were sacrificed when tumor size reached 12 mm in either dimension.

Flow cytometry

For flow cytometry analysis, cells were pre-incubated with α-CD16/32 (clone 2.4G2) to block Fc receptors and then surface stained using antibodies against CD4 (RM4-5 or GK1.5), CD8 (53-6.7), CD11c (N418), CD25 (PC61), CD44 (IM7), CD45 (30-F11), CD69 (H1.2F3), CD71 (R17217), B220 (RA3-6B2), NK1.1 (PK136), F4/80 (BM8), GITR (DTA1), PD-1 (RMP1-30 or 29F.1A12), TCRβ (H57-597), and TCR γδ (GL3). Antibodies were purchased from eBioscience (now Thermo Fisher) or Biolegend. Stainings were performed with appropriate combinations of fluorophores or streptavidin conjugated fluorophores. Propidium iodide (ImmunoChemistry Technologies) or 4′,6-diamidino-2-phenylindole (DAPI) was used in some analyses to distinguish live and dead cells. Data were acquired with FACSCanto I or BD LSRFortessa flow cytometers and analyzed using the FlowJo software. For the analysis of thymocytes, a lineage cocktail was used, including: B220, NK1.1, F4/80, TCR γδ, CD11c.

Cytokine production/degranulation capacity assay/Foxp3 staining

Tumor cell suspensions (cultured in RPMI 1640 supplemented with 10% FCS, 100 U/ml penicillin, 100 μg/ml streptomycin, 1 mM sodium pyruvate, 50 μM 2-mercaptoethanol and 10 mM HEPES buffer (all from Life technologies) at 37°C with 5% CO^2) were stimulated with 10 nM PMA (Millipore) and 1 μg/ml Ionomycin (Sigma) for 1 h followed by 3 h with 5 μg/ml of Brefeldin A (Sigma). After stimulation, cells were stained extracellularly as described above, fixed and permeabilized using the Transcription factor fixation/permeabilization buffer from eBioscience following the recommended protocol. After permeabilization, cells were then stained for the indicated cytokines (TNF-α clone MP6-XT22, IFN-γ clone XMG1.2). The same fixation/permeabilization protocol was used for Foxp3 (FJK-16s) staining of *ex vivo* cells.

CTL differentiation

T cells were activated and cultured at 37°C with 5% CO2 in RPMI 1640 containing glutamine (Invitrogen), supplemented with 10% FBS (Gibco), penicillin/streptomycin (Gibco), and 50 μM β-mercaptoethanol (Sigma) unless otherwise indicated. Spleen was homogenized, red blood cells lysed, and $CD4^+$ T cells depleted using the EasySep Mouse Streptavidin RapidSpheres Isolation Kit from StemCell Technologies. Cells were then cultured at a cell density of 2 million/ml in the presence of CD3 (2C11; 1 μg/ml) and CD28 (37.51; 0.5 μg/ml) antibodies supplemented with 20 ng/ml recombinant human IL-2 (Proleukin, Novartis) and 2 ng/ml recombinant mouse IL-12 (Peprotech) for 48 h. Then, cells were washed and further cultured in media with 20 ng/ml IL-2, regularly splitting them to 0.3×10^6/ml.

Proteomics sample preparation

Peptide extraction and fractionation and LC–MS analysis was performed by the FingerPrints Proteomics Facility (University of Dundee). Cell pellets were processed using S-trap mini protocol (Protifi) as recommended by the manufacturer with little modification. After application of the samples on the S-trap mini spin column, trapped proteins were washed five times with S-TRAP binding buffer. Digestion with trypsin (10 μg) was carried out overnight at 37°C in 160 μl of TEAB at a final concentration of 50 mM. Elution of peptides from S-trap mini spin column was achieved by centrifugation by adding 160 μl of 50 mM ammonium bicarbonate, then 160 μl of 2% aqueous formic acid and finally 160 μl of 50% acetonitrile/0.2% formic acid. Resulting tryptic peptides were dried and quantified using Pierce Quantitative fluorometric Peptide Assay (Thermo Scientific).

High pH RP fractionation

Peptides (77 μg each sample) were resuspended in 200 μl buffer A (10 mM ammonium formate in milliQ water pH 9) and then fractionated with an Ultimate 3000 HPLC system (Thermo-Scientific) (High pH RP Chromatography). A C18 Column from Waters (XBridge peptide BEH, 130 Å, 3.5 μm 2.1 × 150 mm, Waters, Ireland) and a guard column (XBridge, C18, 3.5 μm, 2.1 × 10 mm, Waters) were used. Fractions were collected using a WPS-3000FC auto-sampler (Thermo-Scientific) at 1-min intervals.

Column and guard column were equilibrated with 2% Buffer B (10 mM ammonium formate, pH 9 in 90% acetonitrile) for 18 min at a constant flow rate of 0.2 ml/min and a constant temperature of 20°C. Samples (190 μl) were loaded onto the column at 0.2 ml/min, and separation gradient started 1 min after sample were loaded onto the column. Peptides were eluted from the column with a gradient of 2% buffer B to 60% B within 19 min, then from 60% B to 100% B in 5 min. The guard and the column were washed for 10 min at 100% buffer B and equilibrated at 2% buffer B for 18 min as mentioned above. A blank was run between each sample using the same conditions. Fraction collection started 1 min after injection and stopped after 40 min. The total number of fractions concatenated was set to 8, and the content of the fractions was dried and suspended in 50 μl 1% formic acid prior to analysis with LC–MS. Fractions 5–8 of each sample were cleaned up using HiPPR detergent removal kit (Thermo Scientific) following manufacturer protocol.

LC–MS/MS analysis

Analysis of peptide readout was performed on a Q Exactive™ plus, Mass Spectrometer (Scientific) coupled with a Dionex Ultimate 3000 RS (Thermo Scientific). LC buffers used are the following: buffer A (0.1% formic acid in Milli-Q water (v/v)) and buffer B (80% acetonitrile and 0.1% formic acid in Milli-Q water (v/v)). Aliquots of 5 μl (equivalent of 1 μg) of each sample were loaded at 10 μl/min onto a trap column (100 μm × 2 cm, PepMap nanoViper C18 column, 5 μm, 100 Å, Thermo Scientific) equilibrated in 0.1% TFA. The trap column was washed for 3 min at the same flow rate with 0.1% TFA and then switched in-line with a Thermo Scientific resolving C18 column (75 μm × 50 cm, PepMap RSLC C18 column, 2 μm, 100 Å). The peptides were eluted from the column at a constant flow rate of 300 nl/min with a linear gradient from 2% buffer B to 5% buffer B in 5 min then from 5% buffer B to 35% buffer B in 125 min, from 35% buffer B to 98% buffer B in 2 min. The column was then washed with 98% buffer B for 20 min and re-equilibrated in 2% buffer B for 17 min. The column was kept at a constant temperature of 50°C.

© 2022 The Authors

Q Exactive™ plus was operated in data dependent positive ionization mode. The source voltage was set to 2.5 Kv and the capillary temperature was 250°C.

A scan cycle comprised MS1 scan (*m/z* range from 350–1,600, ion injection time of 20 ms, resolution 70,000 and automatic gain control (AGC) 1×10^6) acquired in profile mode, followed by 15 sequential dependent MS2 scans (resolution 17,500) of the most intense ions fulfilling predefined selection criteria (AGC 2×10^5, maximum ion injection time 100 ms, isolation window of 1.4 *m/z*, fixed first mass of 100 *m/z*, spectrum data type: centroid, intensity threshold 2×10^4, exclusion of unassigned, singly and > 6 charged precursors, peptide match preferred, exclude isotopes on, dynamic exclusion time 45 s). The HCD collision energy was set to 27% of the normalized collision energy. Mass accuracy is checked before the start of samples analysis.

Processing and analysis of proteomic data

The data were processed, searched, and quantified with the MaxQuant software package (version 1.6.10.43). Proteins and peptides were identified using a hybrid database from databases in Uniprot release 2020 06 as described in Marchingo *et al* (2020). The following search parameters were used: protein N-terminal acetylation, methionine oxidation, glutamine to pyroglutamate, and glutamine and asparagine deamidation were selected as variable modifications; carbamidomethylation of cysteine residues was set as a fixed modification; Trypsin and LysC were selected as the proteolytic enzymes; up to two missed cleavages were permitted; protein and PSM False discovery rates was set to 0.01 and matching of peptides between runs was switched off. Perseus software package (version 1.6.6.0) was used for data filtering and protein copy number quantification. Proteins were quantified from unique (found only in a specific protein group) and razor (peptides assigned to a specific protein group without being unique to that group) peptides. The data set was filtered to remove proteins categorized as "contaminants", "reverse" and "only identified by site". Copy numbers were calculated using the proteomic ruler plugin as previously described (Wisniewski *et al*, 2014). Copy numbers of histones in a diploid mouse cell get assigned to the summed peptide intensities of all histones present in a sample. The ratio between the histone peptide intensity and the summed peptide intensities of the other identified proteins is then used to estimate copy number per cell for all identified proteins in the data set.

Statistics and calculations of proteomic data

Three biological replicates were generated. *P*-values were calculated via a two-tailed, unequal-variance *t*-test on $\log_{10}$ transformed copy number per cell values in Microsoft Excel. *P*-values < 0.05 were considered as being statistically significant. Fold change > 1.5 or < 0.67 were considered as cut-off.

GO term analysis of proteomic data

Pathway analysis of significantly downregulated genes in Ptpn6/11-deleted CTLs and of genes expressed only in control CTLs was performed with Database for Annotation, Visualization and Integrated Discovery (DAVID) bioinformatics tool with GOTERM BP Direct database (https://david.ncifcrf.gov).

Cell cycle analysis

Cells were washed and fixed with cold 70% ethyl alcohol for 30 min. Cells were then washed with cold PBS and incubated with 200 µg/ml RNase A for 30 min at 37°C. Cells were then stained with 100 µg/ml propidium iodide for 30 min at room temperature and analyzed by flow cytometry.

Western blot

T cells were enriched by negative selection using the EasySep™ Mouse T Cell Isolation Kit (STEMCELL Technologies) following the recommended protocol. T cell purity was over 90%. T cells were then resuspended in sample buffer (250 mM NaCl, 50 mM HEPES, pH 7.5, 1% NP-40, 5 mM EDTA) supplemented with protease inhibitor cocktail and phosphatase inhibitor (Roche) for protein lysis and extraction. Protein concentrations were assessed by Bradford assay. Rabbit monoclonal antibodies against Ptpn11 (#33975 clone D50F2), Ptpn6 (#3759 clone C14H6) were used for immunoblotting. The anti-Gapdh antibody (#G9545 polyclonal) was used as control for the experiments.

Statistical analysis

Unless otherwise specified, statistical analyses were performed using Prism software (GraphPad v.5.0). Student's *t*-test (unpaired, two-tailed) was used to compare differences between experimental groups. Differences were considered significant when $*P < 0.05$, very significant when $**P < 0.01$, and highly significant when $***P < 0.001$ and $****P \leq 0.0001$. For survival, comparisons are by log-rank (Mantel-Cox) test.

Data availability

The mass spectrometry proteomics dataset produced in this study is available at the PRIDE partner repository with the dataset identifier PXD034897: https://www.ebi.ac.uk/pride/archive?keyword=PXD034897.

Expanded View for this article is available online.

Acknowledgements
We thank Irene Buzzago, David Jarrossay, Matteo Pecoraro, and the Animal Facility collaborators for precious help and technical advice at the IRB, Bellinzona, and Douglas Fearon, Cambridge, and Eric Vivier, Marseille, for reagents and advice. We thank E. Emslie for technical assistance with mouse genotyping, A. Gardner, A. Rennie, and R. Clarke from the Flow Cytometry facility for cell sorting, M. Gierlinski for help with statistical analysis, the Fingerprint proteomics facility for the mass spectrometry and advice on proteomics, and the Biological Services Resource unit at the University of Dundee. Graphical abstract and some figure panels were created with Biorender. This work was supported by the Swiss National Science Foundation (310030_185185 and 310030_197771 to GG), the Fondazione San Salvatore, Lugano, the Swiss Cancer Research Foundation (KFS 5141-08-2020 to GG), the Leonardo Foundation, Lugano, the Novartis Foundation, Basel. This research was supported by a Wellcome Trust Principal Research Fellowship to DAC (205023/Z/16/Z).

© 2022 The Authors

Author contributions

Pedro MO Ventura: Conceptualization; data curation; formal analysis; investigation; writing – original draft; writing – review and editing. **Milica Gakovic:** Conceptualization; data curation; formal analysis; investigation; writing – original draft; writing – review and editing. **Berenice A Fischer:** Investigation. **Laura Spinelli:** Data curation; investigation. **Giorgia Rota:** Investigation. **Shalini Pathak:** Investigation. **Hanif J Khameneh:** Investigation. **Alessandro Zenobi:** Investigation. **Sarah Thomson:** Investigation. **Doreen A Cantrell:** Conceptualization; supervision; funding acquisition; writing – original draft; writing – review and editing. **Greta Guarda:** Conceptualization; supervision; funding acquisition; writing – original draft; writing – review and editing.

Disclosure and competing interests statement

Unrelated projects in GG laboratory are supported by OM-Pharma, Meyrin, and IFM Therapeutics, Boston. DAC has collaborations with GlaxoSmithKline and Division of Signal Transduction Therapy (DSTT), involving collaborations with Merck, Boehringer Ingleheim, and GSK. The other authors declare no competing interests.

References

Calzascia T, Pellegrini M, Hall H, Sabbagh L, Ono N, Elford AR, Mak TW, Ohashi PS (2007) TNF-alpha is critical for antitumor but not antiviral T cell immunity in mice. *J Clin Invest* 117: 3833 – 3845

Carrington EM, Zhan Y, Brady JL, Zhang JG, Sutherland RM, Anstee NS, Schenk RL, Vikstrom IB, Delconte RB, Segal D *et al* (2017) Anti-apoptotic proteins BCL-2, MCL-1 and A1 summate collectively to maintain survival of immune cell populations both *in vitro* and *in vivo*. *Cell Death Differ* 24: 878 – 888

Celis-Gutierrez J, Blattmann P, Zhai Y, Jarmuzynski N, Ruminski K, Gregoire C, Ounoughene Y, Fiore F, Aebersold R, Roncagalli R *et al* (2019) Quantitative interactomics in primary T cells provides a rationale for concomitant PD-1 and BTLA coinhibitor blockade in cancer immunotherapy. *Cell Rep* 27: 3315 – 3330

Chemnitz JM, Parry RV, Nichols KE, June CH, Riley JL (2004) SHP-1 and SHP-2 associate with immunoreceptor tyrosine-based switch motif of programmed death 1 upon primary human T cell stimulation, but only receptor ligation prevents T cell activation. *J Immunol* 173: 945 – 954

Dance M, Montagner A, Salles JP, Yart A, Raynal P (2008) The molecular functions of Shp2 in the Ras/Mitogen-activated protein kinase (ERK1/2) pathway. *Cell Signal* 20: 453 – 459

Dzhagalov I, Dunkle A, He YW (2008) The anti-apoptotic Bcl-2 family member Mcl-1 promotes T lymphocyte survival at multiple stages. *J Immunol* 181: 521 – 528

Fowler CC, Pao LI, Blattman JN, Greenberg PD (2010) SHP-1 in T cells limits the production of CD8 effector cells without impacting the formation of long-lived central memory cells. *J Immunol* 185: 3256 – 3267

Gaud G, Lesourne R, Love PE (2018) Regulatory mechanisms in T cell receptor signalling. *Nat Rev Immunol* 18: 485 – 497

Grossmann KS, Wende H, Paul FE, Cheret C, Garratt AN, Zurborg S, Feinberg K, Besser D, Schulz H, Peles E *et al* (2009) The tyrosine phosphatase Shp2 (PTPN11) directs Neuregulin-1/ErbB signaling throughout Schwann cell development. *Proc Natl Acad Sci USA* 106: 16704 – 16709

Hebeisen M, Baitsch L, Presotto D, Baumgaertner P, Romero P, Michielin O, Speiser DE, Rufer N (2013) SHP-1 phosphatase activity counteracts increased T cell receptor affinity. *J Clin Invest* 123: 1044 – 1056

Hui E, Cheung J, Zhu J, Su X, Taylor MJ, Wallweber HA, Sasmal DK, Huang J, Kim JM, Mellman I *et al* (2017) T cell costimulatory receptor CD28 is a primary target for PD-1-mediated inhibition. *Science* 355: 1428 – 1433

Iype T, Sankarshanan M, Mauldin IS, Mullins DW, Lorenz U (2010) The protein tyrosine phosphatase SHP-1 modulates the suppressive activity of regulatory T cells. *J Immunol* 185: 6115 – 6127

Jacob J, Baltimore D (1999) Modelling T-cell memory by genetic marking of memory T cells *in vivo*. *Nature* 399: 593 – 597

Johnson DJ, Pao LI, Dhanji S, Murakami K, Ohashi PS, Neel BG (2013) Shp1 regulates T cell homeostasis by limiting IL-4 signals. *J Exp Med* 210: 1419 – 1431

Juneja VR, McGuire KA, Manguso RT, LaFleur MW, Collins N, Haining WN, Freeman GJ, Sharpe AH (2017) PD-L1 on tumor cells is sufficient for immune evasion in immunogenic tumors and inhibits CD8 T cell cytotoxicity. *J Exp Med* 214: 895 – 904

Kamphorst AO, Wieland A, Nasti T, Yang S, Zhang R, Barber DL, Konieczny BT, Daugherty CZ, Koenig L, Yu K *et al* (2017) Rescue of exhausted CD8 T cells by PD-1-targeted therapies is CD28-dependent. *Science* 355: 1423 – 1427

Kim EY, Teh HS (2004) Critical role of TNF receptor type-2 (p75) as a costimulator for IL-2 induction and T cell survival: a functional link to CD28. *J Immunol* 173: 4500 – 4509

Marasco M, Berteotti A, Weyershaeuser J, Thorausch N, Sikorska J, Krausze J, Brandt HJ, Kirkpatrick J, Rios P, Schamel WW *et al* (2020) Molecular mechanism of SHP2 activation by PD-1 stimulation. *Sci Adv* 6: eaay4458

Marchingo JM, Sinclair LV, Howden AJ, Cantrell DA (2020) Quantitative analysis of how Myc controls T cell proteomes and metabolic pathways during T cell activation. *Elife* 9: e53725

Martinez RJ, Morris AB, Neeld DK, Evavold BD (2016) Targeted loss of SHP1 in murine thymocytes dampens TCR signaling late in selection. *Eur J Immunol* 46: 2103 – 2110

Mullard A (2018) Phosphatases start shedding their stigma of undruggability. *Nat Rev Drug Discov* 17: 847 – 849

Niogret C, Birchmeier W, Guarda G (2019a) SHP-2 in lymphocytes' cytokine and inhibitory receptor signaling. *Front Immunol* 10: 2468

Niogret C, Miah SMS, Rota G, Fonta NP, Wang H, Held W, Birchmeier W, Sexl V, Yang W, Vivier E *et al* (2019b) Shp-2 is critical for ERK and metabolic engagement downstream of IL-15 receptor in NK cells. *Nat Commun* 10: 1444

Nishida M, Yamashita N, Ogawa T, Koseki K, Warabi E, Ohue T, Komatsu M, Matsushita H, Kakimi K, Kawakami E *et al* (2021) Mitochondrial reactive oxygen species trigger metformin-dependent antitumor immunity via activation of Nrf2/mTORC1/p62 axis in tumor-infiltrating CD8T lymphocytes. *J Immunother Cancer* 9: e002954

Poole AW, Jones ML (2005) A SHPing tale: perspectives on the regulation of SHP-1 and SHP-2 tyrosine phosphatases by the C-terminal tail. *Cell Signal* 17: 1323 – 1332

Rafiq S, Hackett CS, Brentjens RJ (2020) Engineering strategies to overcome the current roadblocks in CAR T cell therapy. *Nat Rev Clin Oncol* 17: 147 – 167

Rota G, Niogret C, Dang AT, Barros CR, Fonta NP, Alfei F, Morgado L, Zehn D, Birchmeier W, Vivier E *et al* (2018) Shp-2 is dispensable for establishing T cell exhaustion and for PD-1 signaling *in vivo*. *Cell Rep* 23: 39 – 49

Sheppard KA, Fitz LJ, Lee JM, Benander C, George JA, Wooters J, Qiu Y, Jussif JM, Carter LL, Wood CR *et al* (2004) PD-1 inhibits T-cell receptor induced phosphorylation of the ZAP70/CD3zeta signalosome and downstream signaling to PKCtheta. *FEBS Lett* 574: 37 – 41

Snook JP, Soedel AJ, Ekiz HA, O'Connell RM, Williams MA (2020) Inhibition of SHP-1 expands the repertoire of antitumor T cells available to respond to immune checkpoint blockade. *Cancer Immunol Res* 8: 506 – 517

© 2022 The Authors

Srinivas S, Watanabe T, Lin CS, William CM, Tanabe Y, Jessell TM, Costantini F (2001) Cre reporter strains produced by targeted insertion of EYFP and ECFP into the ROSA26 locus. *BMC Dev Biol* 1: 4

Strauss L, Mahmoud MAA, Weaver JD, Tijaro-Ovalle NM, Christofides A, Wang Q, Pal R, Yuan M, Asara J, Patsoukis N *et al* (2020) Targeted deletion of PD-1 in myeloid cells induces antitumor immunity. *Sci Immunol* 5: eaay1863

Stromnes IM, Fowler C, Casamina CC, Georgopolos CM, McAfee MS, Schmitt TM, Tan X, Kim TD, Choi I, Blattman JN *et al* (2012) Abrogation of SRC homology region 2 domain-containing phosphatase 1 in tumor-specific T cells improves efficacy of adoptive immunotherapy by enhancing the effector function and accumulation of short-lived effector T cells *in vivo*. *J Immunol* 189: 1812 – 1825

Topalian SL (2017) Targeting immune checkpoints in cancer therapy. *JAMA* 318: 1647 – 1648

Varone A, Spano D, Corda D (2020) Shp1 in solid cancers and their therapy. *Front Oncol* 10: 935

Viant C, Fenis A, Chicanne G, Payrastre B, Ugolini S, Vivier E (2014) SHP-1-mediated inhibitory signals promote responsiveness and anti-tumour functions of natural killer cells. *Nat Commun* 5: 5108

Wherry EJ, Kurachi M (2015) Molecular and cellular insights into T cell exhaustion. *Nat Rev Immunol* 15: 486 – 499

Wisniewski JR, Hein MY, Cox J, Mann M (2014) A “proteomic ruler” for protein copy number and concentration estimation without spike-in standards. *Mol Cell Proteomics* 13: 3497 – 3506

Xu X, Hou B, Fulzele A, Masubuchi T, Zhao Y, Wu Z, Hu Y, Jiang Y, Ma Y, Wang H *et al* (2020) PD-1 and BTLA regulate T cell signaling differentially and only partially through SHP1 and SHP2. *J Cell Biol* 219: e201905085

Yokosuka T, Takamatsu M, Kobayashi-Imanishi W, Hashimoto-Tane A, Azuma M, Saito T (2012) Programmed cell death 1 forms negative costimulatory microclusters that directly inhibit T cell receptor signaling by recruiting phosphatase SHP2. *J Exp Med* 209: 1201 – 1217

Yuan X, Bu H, Zhou J, Yang CY, Zhang H (2020) Recent advances of SHP2 inhibitors in cancer therapy: current development and clinical application. *J Med Chem* 63: 11368 – 11396

Zhang J, Somani AK, Yuen D, Yang Y, Love PE, Siminovitch KA (1999) Involvement of the SHP-1 tyrosine phosphatase in regulation of T cell selection. *J Immunol* 163: 3012 – 3021

Zhang SQ, Yang W, Kontaridis MI, Bivona TG, Wen G, Araki T, Luo J, Thompson JA, Schraven BL, Philips MR *et al* (2004) Shp2 regulates SRC family kinase activity and Ras/Erk activation by controlling Csk recruitment. *Mol Cell* 13: 341 – 355

License: This is an open access article under the terms of the Creative Commons Attribution License, which permits use, distribution and reproduction in any medium, provided the original work is properly cited.

Expanded View Figures

Figure EV1. Peripheral T cell compartment characterization of *CD4cre Ptpn6*$^{fl/fl}$ and *CD4cre Ptpn6*$^{fl/fl}$/*Ptpn11*$^{fl/fl}$ mice.

A, B T cells from spleen (A) and inguinal lymph nodes (iLN) (B) of *CD4cre Ptpn6*$^{fl/fl}$ and control mice were analyzed. Cellularity, $CD4^+$ and $CD8^+$ T cell numbers (gated as TCRβ$^+$ $CD4^+$ or $CD8^{+,}$ respectively), percentages of CD44 high T cells are shown; regulatory T cell (Treg) numbers and frequency (gated as TCRβ$^+$ $CD4^+$ Foxp3$^+$) are illustrated for the spleen.

C Cellularity from thymus and number of CD4 and CD8 double-negative, double-positive, CD4 or CD8 single-positive thymocytes (gated on lineage$^-$; single-positives, an additional gate on TCRβ$^+$ was performed) of *CD4cre Ptpn6*$^{fl/fl}$/*Ptpn11*$^{fl/flWbm}$ and control mice are depicted.

D Treg number and frequency (gated as TCRβ$^+$ $CD4^+$ Foxp3$^+$) are illustrated for the spleen of *CD4cre Ptpn6*$^{fl/fl}$/*Ptpn11*$^{fl/flWbm}$ and control mice.

E Cellularity, $CD4^+$ and $CD8^+$ T cell numbers, as well as percentages of CD44 high T cells from the iLN of *CD4cre Ptpn6*$^{fl/fl}$/*Ptpn11*$^{fl/flWbm}$ and control mice are shown.

F Survival curves of *CD4cre Ptpn6*$^{fl/fl}$/*Ptpn11*$^{fl/flBgn}$ and control mice challenged with MC38.

G–I Ten to twelve days following MC38 tumor inoculation, isotype-treated *CD4cre Ptpn6/11*$^{wt/wt}$ and *CD4cre Ptpn6*$^{fl/fl}$/*Ptpn11*$^{fl/flWbm}$ mice were sacrificed. Graph depicts the frequencies of PD-1$^+$ $CD8^+$ T cells (gated as $CD45^+$ TCRβ$^+$ $CD8^+$) in the tumor (G). Frequencies of $CD4^+$ T cells (gated as $CD45^+$ TCRβ$^+$ $CD4^+$) expressing IFN-γ and TNFα upon re-stimulation (H) and percentages of $CD4^+$ T cells in the tumor are shown (I).

Data information: Results illustrate mean ± SEM of n = 7–8 mice/group (A, B), of n = 10–12 mice/group (C–E), or of n = 9–14 mice/group (G–I). Student's t-test (unpaired, two-tailed) was used to compare differences between experimental groups (A–E, G–I). Results depict n = 11 mice/group; statistical significance was calculated by log-rank (Mantel-Cox) test (F). *$P \leq 0.05$, **$P \leq 0.01$, ***$P \leq 0.001$, ****$P \leq 0.0001$.
Source data are available online for this figure.

 © 2022 The Authors

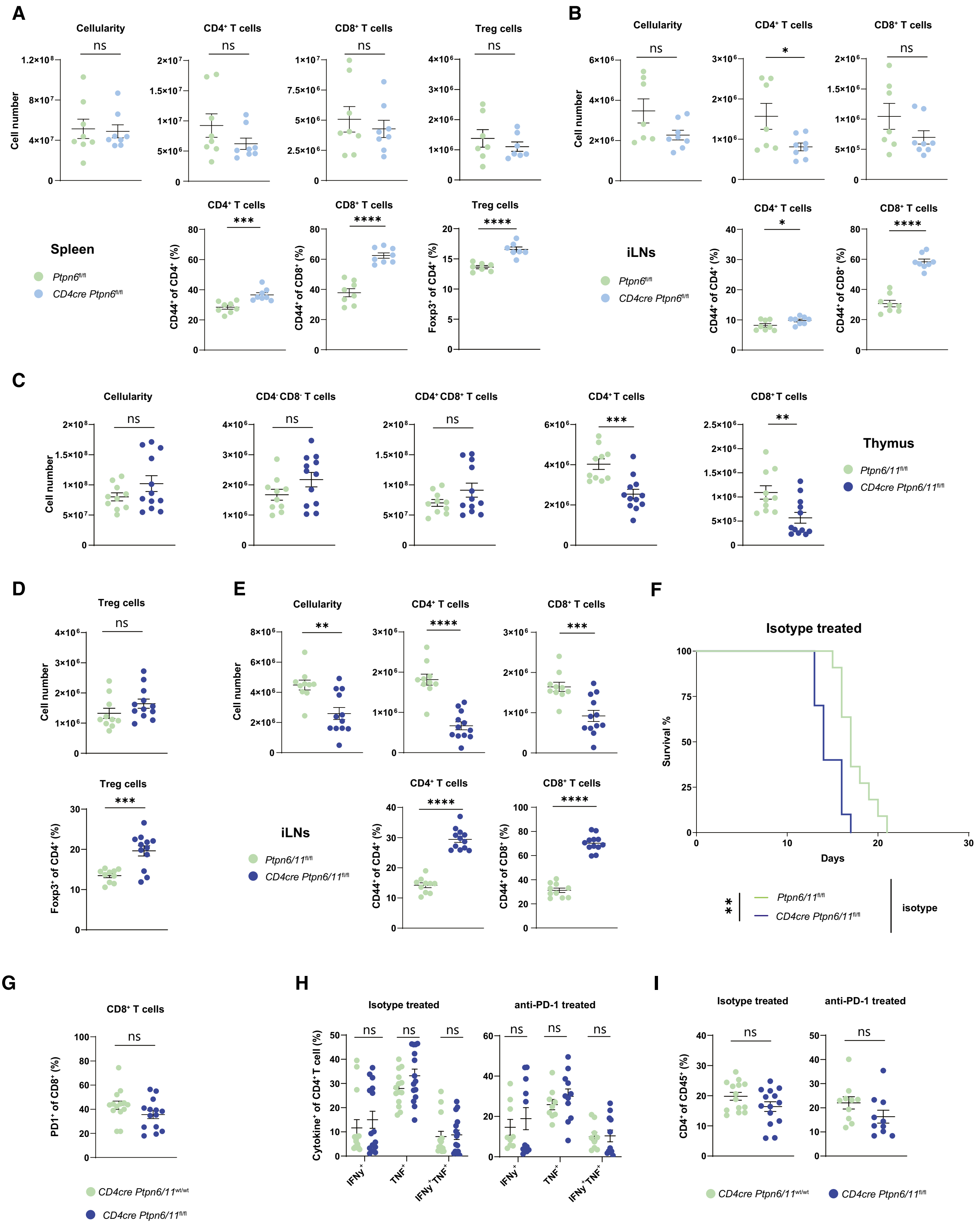
A
Cellularity
CD4+ T cells
CD8+ T cells
Treg cells
Cell number
ns
CD44+ of CD4+ (%)
CD44+ of CD8+ (%)
Foxp3+ of CD4+ (%)
***
****
Spleen
Ptpn6fl/fl
CD4cre Ptpn6fl/fl
B
iLNs
*
C
CD4-CD8- T cells
CD4+CD8+ T cells
**
Thymus
Ptpn6/11fl/fl
CD4cre Ptpn6/11fl/fl
D
E
F
Isotype treated
Survival %
Days
isotype
G
PD1+ of CD8+ (%)
CD4cre Ptpn6/11wt/wt
H
anti-PD-1 treated
Cytokine+ of CD4+ T cell (%)
IFNγ+
TNF+
IFNγ+TNF+
I
CD4+ of CD45+ (%)

Figure EV1.

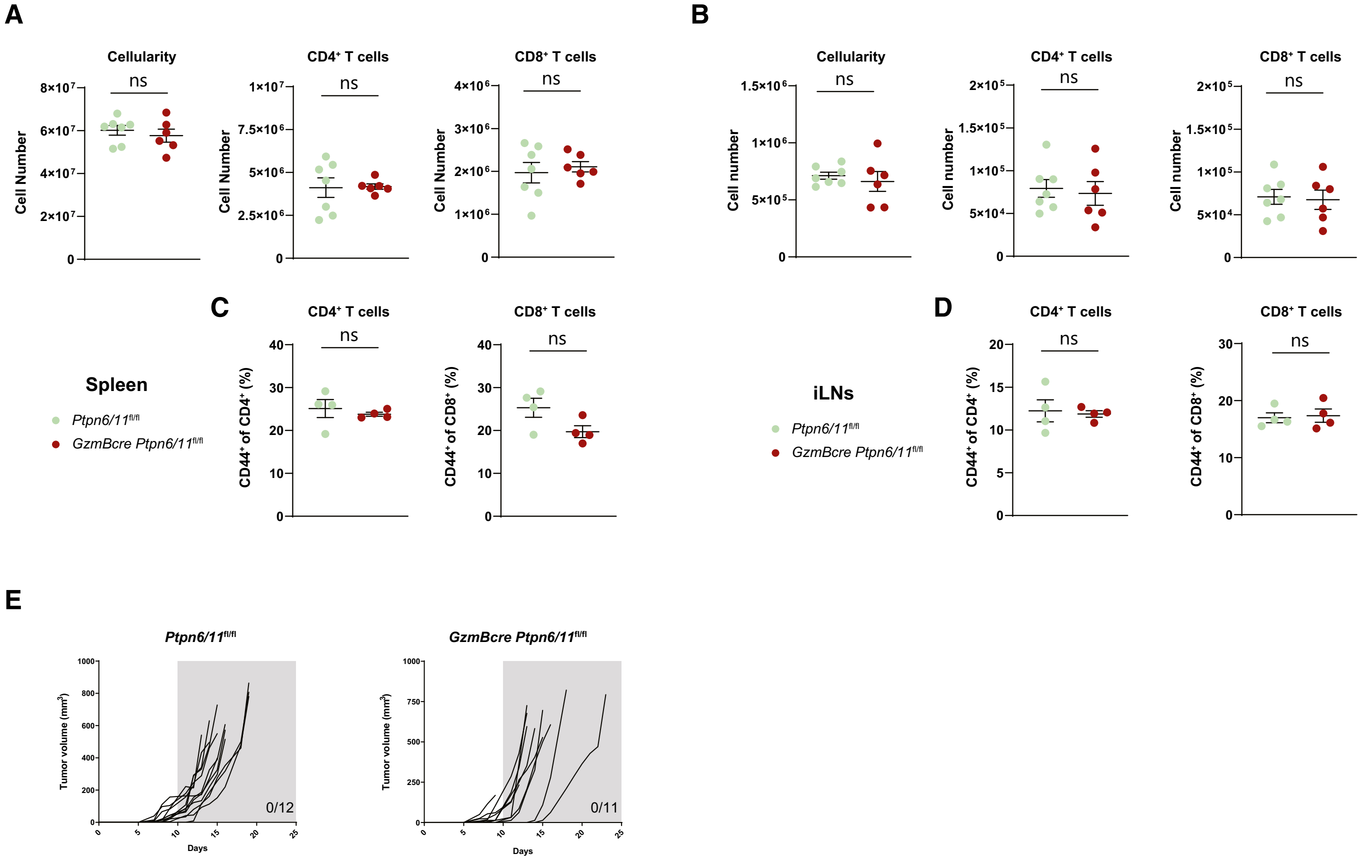

Figure EV2. Peripheral T cell compartment characterization of *GzmBcre Ptpn6/11$^{fl/fl}$* mice.

A–D Spleen and iLN of *GzmBcre Ptpn6/11$^{fl/fl}$* mice and control mice were analyzed. Cellularity, $CD4^+$ and $CD8^+$ T cell numbers are depicted for spleen (A) and iLN (B). Percentages of CD44 high of $CD4^+$ and $CD8^+$ T cells are shown for spleen (C) and iLN (D). Results depict $n = 6–7$ mice/group (A, B) and $n = 4$ mice/group for percentages of CD44 high cells (C, D). Student's *t*-test (unpaired, two-tailed) was used to compare differences between experimental groups.

E Tumor growth in individual mice challenged with MC38 is shown for the indicated genotypes; number of mice eradicating the tumor is shown within the graphs.

Source data are available online for this figure.

 © 2022 The Authors

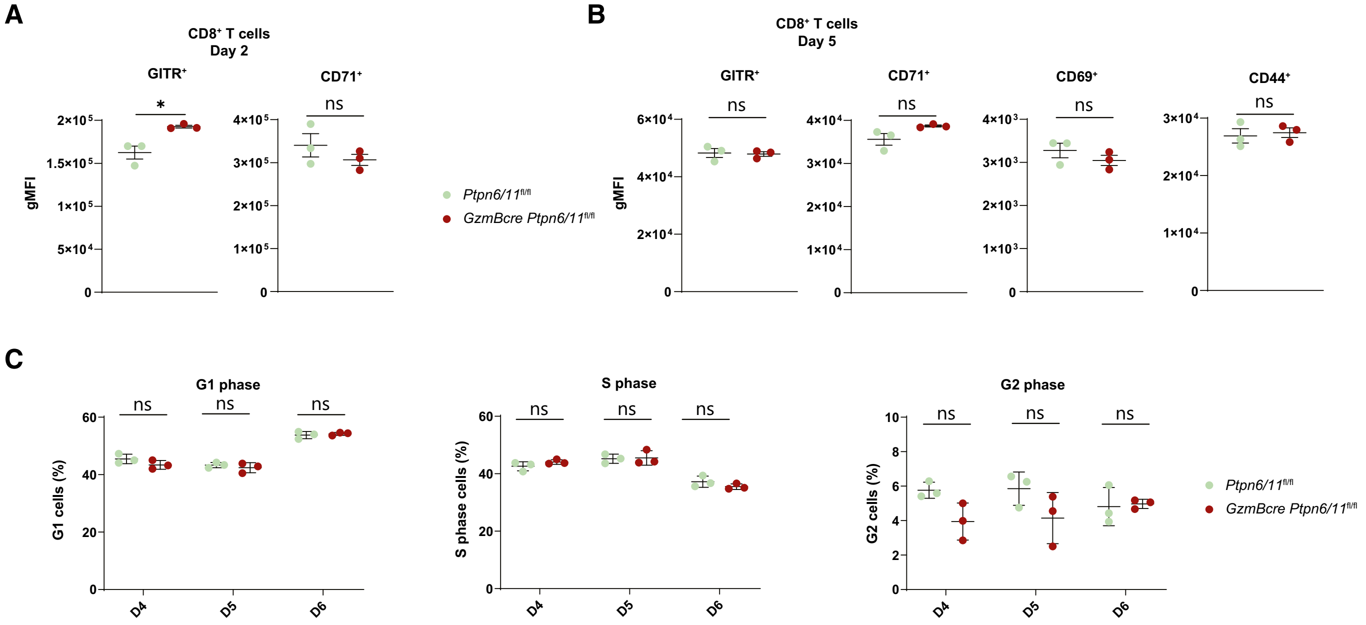

Figure EV3. Characterization of CTLs from *Ptpn6/11*$^{fl/fl}$ and *GzmBcre Ptpn6/11*$^{fl/fl}$ mice.

$CD8^+$ cytotoxic lymphocytes were generated from splenocytes of the indicated mice by anti-CD3, anti-CD28, and IL-12 stimulation and maintained in IL-2.

A, B $CD8^+$ T cells were analyzed by flow cytometry for surface expression of GITR and CD71 after 2 days (A) and for surface expression of GITR, CD71, CD69, and CD44 after 5 days (B); a quantification of these parameters is shown in the graphs (A and B).

C Graphs depict the percentages of cells in G1, S, and G2 phase as measured by flow cytometry-based cell cycle analysis of *Ptpn6/11*$^{fl/fl}$ and *GzmBcre Ptpn6/11*$^{fl/fl}$ CTLs (gated on $CD8^+$ T cells) at the indicated days.

Data information: Results depict mean ± SD of $n = 3$ biological replicates and Student's t-test (unpaired, two-tailed) was used to compare differences between experimental groups (A–C). $^*P \leq 0.05$.
Source data are available online for this figure.

© 2022 The Authors
