# Supplementary material for: Concomitant deletion of Ptpn6 and Ptpn11 in T cells fails to improve anticancer responses
Source: EMBO Rep. 2022 Oct 4;23(11):e55399. doi: 10.15252/embr.202255399 (PMC9638855; doi:10.15252/embr.202255399)
Supplement: Supplementary file 5 — Source Data for Figure 1 [file EMBR-23-e55399-s007.pdf]

Fig 1B - Deletion check western blot

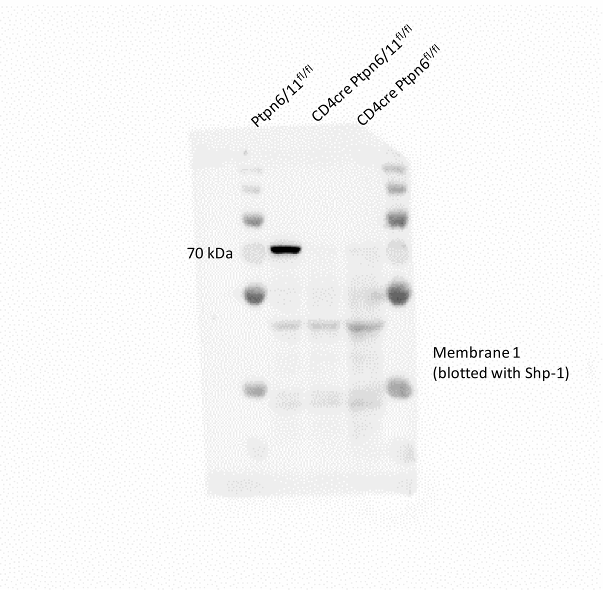

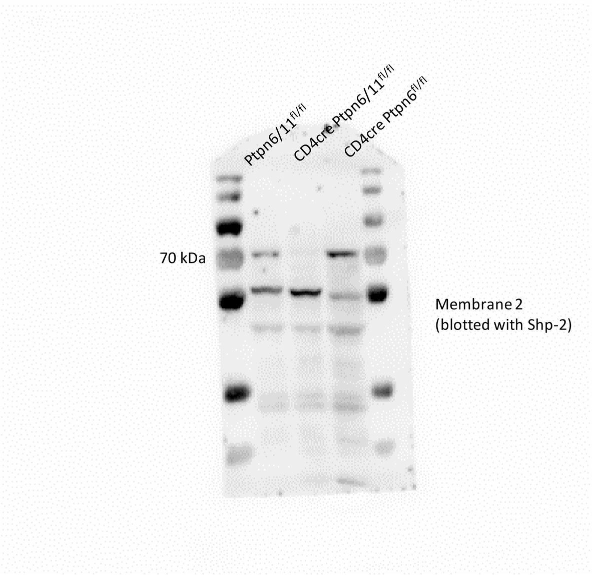

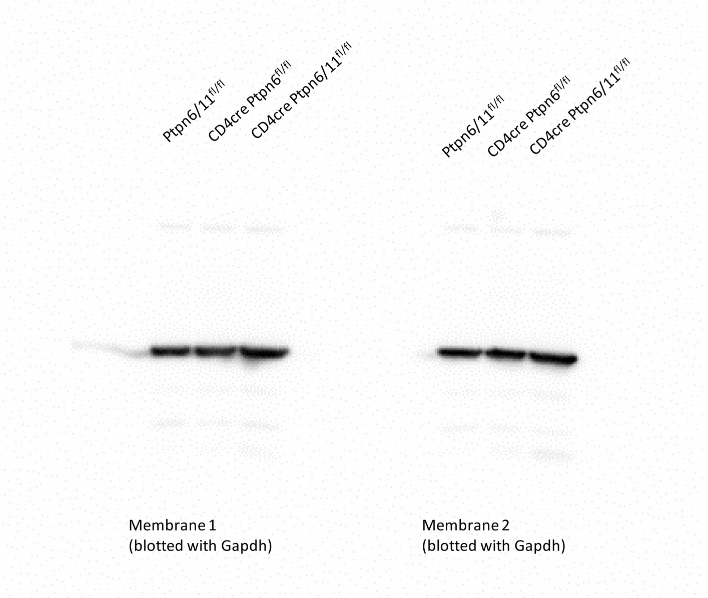

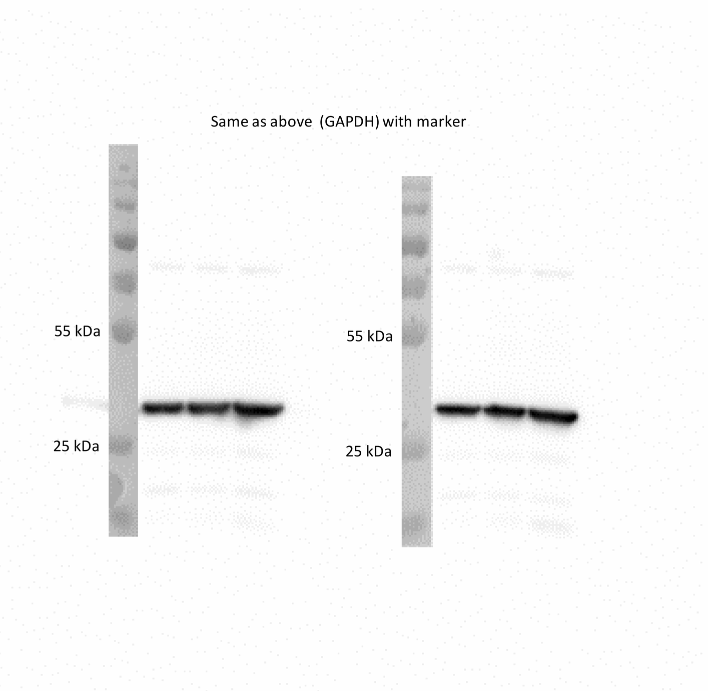

Fig 1D - Survival curve

Days	Ptpn6$^{fl/fl}$ Iso	Ptpn6$^{fl/fl}$ aPD1	CD4cre Ptpn6$^{fl/fl}$ Iso	CD4cre Ptpn6$^{fl/fl}$ aPD1
**18**	1		1	
**21**			1	
**21**			1	
**21**			1	
**21**			1	
**21**			1	
**22**	1		1	1
**22**	1		1	1
**22**	1			
**24**	1			1
**25**	1			1
**25**	1			
**28**	1		1	1
**28**			1	
**30**				1
**30**				1
**32**		1		1
**33**		1		
**40**		1		1
**40**		0		1
**40**		0		
**40**		0		
**40**		0		
**40**		0		
**40**		0		

Fig 1 F - Spleen

	Ptpn6/11$^{fl/fl}$	CD4cre Ptpn6/11$^{fl/fl}$
	32750000	60500000
	60250000	38375000
	52000000	90000000
	47000000	56500000
	60250000	45750000
**Cellularity**	61250000	61750000
	60875000	36750000
	53375000	70000000
	35875000	51375000
	34250000	42375000
		54625000
		41375000
	**Ptpn6/11$^{fl/fl}$**	**CD4cre Ptpn6/11$^{fl/fl}$**
	4984878	6569877
	7432079	4609797
	7898800	10791720
	9277800	6829438
	8620570	5530031
**CD4 numbers**	12284545	6562481
	10804095	5108103
	8071795	9711380
	6280349	8256990
	5178292	5906736
		6810099
		5793079
	**Ptpn6/11$^{fl/fl}$**	**CD4cre Ptpn6/11$^{fl/fl}$**
	4001199	6069360
	6473742	3837807
	6204120	7699104
	5978447	6389811
	6947488	3834463
**CD8 numbers**	8198251	6971143
	7997514	4256532
	6289390	6548850
	4300516	7238943
	5552610	4703625
		7038595
		5829034
	**Ptpn6/11$^{fl/fl}$**	**CD4cre Ptpn6/11$^{fl/fl}$**
	43.5	62.2
	36.1	53.5
	42.7	56.8
	46.3	58.2
	47.7	62.2
	33.05	71.9
**CD4 CD44exp**		
	28.42	58.18
	25.74	49.8
	32.1	56.3
	23.9	52
		54.8
		41.7
	**Ptpn6/11$^{fl/fl}$**	**CD4cre Ptpn6/11$^{fl/fl}$**
	42.9	77.6
	33.4	67.5
	43.9	77
	56.3	81.7
	45	78.7
**CD8 CD44exp**	43.6	88.6
	37	82.2
	39.2	70.6
	39.1	76.7
	39.5	78.4
		85.9
		81.9

Fig 1H - Survival curve

Days	Ptpn6/11$^{fl/fl}$ Iso	Ptpn6/11$^{fl/fl}$ aPD1	CD4cre Ptpn6/11$^{fl/fl}$ Iso	CD4cre Ptpn6/11$^{fl/fl}$ aPD1
**13**	1	1	1	1
**13**			1	1
**15**			1	1
**15**			1	
**16**	1		1	1
**16**			1	
**17**			1	
**17**			1	
**18**	1			
**19**	1	1	1	1
**19**			1	1
**21**	1			1
**21**	1		1	1
**22**	1			1
**22**	1			
**27**				1
**29**		1		
**30**		1		
**30**		1		
**35**		0		
**35**		0		
**35**		0		
**35**		0		
**35**		0		

Fig 1I - Tumor cytokines

	CD4cre Ptpn6/11$^{wt/wt}$ Iso	CD4cre Ptpn6/11$^{wt/wt}$ aPD1	CD4cre Ptpn6/11$^{fl/fl}$ Iso	CD4cre Ptpn6/11$^{fl/fl}$ aPD1
	11.7	28.8	12.6	11.2
	9.6	23	7.3	11.5
	24	22.9	9.1	14.1
	15	23.5	12.4	7.1
	23.3	28	13.4	39.5
	22.4	37.3	27.7	44.9
	15.2	30.2	34.9	27.8
**IFN-y+**	38.4	27.9	28.8	32.5
	25.8	26	32.2	45
	36.1		26	23.5
	8.6		37.3	36.3
	23.1		22.5	
	14.4		30.2	
	11.7		34.9	
			27.5	
	15.88	29.4	19.63	7.47
	10.02	26.4	16.55	9.99
	25.7	21.19	8.12	10.94
	20.88	20.11	11.24	4.03
	21.27	24.42	16.22	39.5
	25.8	28.12	23.16	31.83
	23.8	17.02	31.98	19.7
**TNF-a+**	29.15	28.7	21.25	31.2
	28.3	24.77	24.2	27.1
	27.5		14.94	29.5
	10.4		28.02	38.57
	20.54		21.99	
	12.21		41.5	
	12.07		38.2	
			31.87	
	6.45	18.3	6.63	4.67
	3.08	11.5	4.85	4.93
	13.7	15.1	1.97	3.78
	8.58	11.6	4.3	1.43
	12.8	19.2	6.97	27.4
	14.4	21.9	14.7	23.6
	10	13	23.6	12.8
**IFN-y+ TNF-a+**	25.6	23.2	13.3	19.5
	16.6	21	20	19.7
	21.6		10.2	16.1
	5.05		20.1	29.6
	18		16.4	
	8.48		25.8	
	9.02		26.8	
			21.9	

Fig 1J - Tumor T cells

	CD4cre Ptpn6/11$^{wt/wt}$ Iso	CD4cre Ptpn6/11$^{wt/wt}$ aPD1	CD4cre Ptpn6/11$^{fl/fl}$ Iso	CD4cre Ptpn6/11$^{fl/fl}$ aPD1
	19.5	37.9	22.9	11
	19.3	41.1	15.3	16.7
	35.6	32.6	13.8	16.8
	17.1	20.7	19.2	17.9
	39.1	37.1	19.8	21
	23.7	37.9	7.01	24.8
**CD8+**	25.1	29.9	22.3	16.3
	25.9	23.5	23.4	27.3
	37.9	32	26.3	27
	28.1	27.3	20.7	33.7
	25.1		18.3	
	28.5		25.1	
	17.6		23.9	
	31.8		19.3	
