# Supplementary material for: Concomitant deletion of Ptpn6 and Ptpn11 in T cells fails to improve anticancer responses
Source: EMBO Rep. 2022 Oct 4;23(11):e55399. doi: 10.15252/embr.202255399 (PMC9638855; doi:10.15252/embr.202255399)
Supplement: Supplementary file 6 — Source Data for Figure 2 [file EMBR-23-e55399-s003.pdf]

Fig 2 B - Proteomics deletion check

	Ptpn6/11$^{fl/fl}$	GzmBcre Ptpn6/11$^{fl/fl}$
**Ptpn6**	106399	0
	87634	12126
	63174	1297

	Ptpn6/11$^{fl/fl}$	GzmBcre Ptpn6/11$^{fl/fl}$
**Ptpn11**	29288	0
	25156	3884
	22144	0

Fig 2 C - YFP tumor

	GzmBcre R26R-YFP
**YFP+ of CD8+ TILs**	57.9
	34
	47.1
	49.7

Fig 2 D - YFP tumor

	GzmBcre R26R-YFP
**PD1+ of YFP+/CD8 + TILs**	90.8
	75.9
	91.1
	89.9

Fig 2 E - Survival of GzmBcre mice

Days	Ptpn6/11$^{fl/fl}$	GzmBcre Ptpn6/11$^{fl/fl}$
**9**		1
**12**		1
**13**	1	1
**13**		1
**13**		1
**14**	1	1
**14**	1	
**15**	1	1
**15**	1	1
**15**	1	
**16**	1	1
**16**	1	
**16**	1	
**18**		1
**19**	1	
**19**	1	
**19**	1	
**23**		1

Fig 2 F - Tumor start and growth rate

	Ptpn6/11$^{fl/fl}$	GzmBcre Ptpn6/11$^{fl/fl}$
**Days passed until tumor detected**	7	5
	7	5
	7	7
	7	7
	7	7
	7	8
	7	9
	7	11
	7	11
	8	14
	8	15
	12	

	Ptpn6/11$^{fl/fl}$	GzmBcre Ptpn6/11$^{fl/fl}$
**Days from tumor detection until experimental endpoint reached**	8	5
	9	8
	10	5
	10	7
	7	9
	13	6
	8	5
	12	6
	9	3
	8	10
	10	9
	8	
