# Supplementary material for: Concomitant deletion of Ptpn6 and Ptpn11 in T cells fails to improve anticancer responses
Source: EMBO Rep. 2022 Oct 4;23(11):e55399. doi: 10.15252/embr.202255399 (PMC9638855; doi:10.15252/embr.202255399)
Supplement: Supplementary file 7 — Source Data for Figure 3 [file EMBR-23-e55399-s006.pdf]

Fig 3 A - Day 2

	Ptpn6/11$^{fl/fl}$	GzmBcre Ptpn6/11$^{fl/fl}$
FSC	2080000	2050000
	2130000	2050000
	2100000	2070000
	**Ptpn6/11$^{fl/fl}$**	**GzmBcre Ptpn6/11$^{fl/fl}$**
SSC	104290	103008
	107930	104142
	105978	104759
	**Ptpn6/11$^{fl/fl}$**	**GzmBcre Ptpn6/11$^{fl/fl}$**
CD25 MFI	1740000	1720000
	1740000	1820000
	2120000	1780000
	**Ptpn6/11$^{fl/fl}$**	**GzmBcre Ptpn6/11$^{fl/fl}$**
PD1 MFI	33210	32079
	35484	34597
	45207	34320

Fig 3 C - CTL expansion

	Ptpn6/11$^{fl/fl}$	GzmBcre Ptpn6/11$^{fl/fl}$
Day 4	1950000	1510000
	1790000	1660000
	1890000	1540000
	1820000	1440000
Day 5	4680000	3550000
	4500000	3500000
	4500000	2950000
	4320000	3500000
Day 6	14040000	8625000
	18900000	9625000
	16560000	7750000
	16020000	8500000
Day 7	59760000	23250000
	66960000	25125000
	60480000	19875000
	55440000	22125000

Fig 3 G - Dead cells

	Ptpn6/11$^{fl/fl}$	GzmBcre Ptpn6/11$^{fl/fl}$
Day 4	20.5	25.9
	20.7	24.5
	18.8	25.4
	20.8	25
Day 5	18.1	27.7
	18.2	23.8
	18.4	27.8
	18.5	25.5
Day 6	15.8	25.4
	14.8	21.5
	15.2	25.2
	14.8	23.4
Day 7	15.5	26.9
	14.1	22.2
	17	25
	15.9	24.9

Fig 3 B - Day 5

	Ptpn6/11$^{fl/fl}$	GzmBcre Ptpn6/11$^{fl/fl}$
FSC	760000	694000
	728000	713000
	728000	714000
	**Ptpn6/11$^{fl/fl}$**	**GzmBcre Ptpn6/11$^{fl/fl}$**
SSC	101703	102665
	99207	99867
	98284	100320
	**Ptpn6/11$^{fl/fl}$**	**GzmBcre Ptpn6/11$^{fl/fl}$**
CD25 MFI	233269	323214
	184535	323537
	224082	309311
	**Ptpn6/11$^{fl/fl}$**	**GzmBcre Ptpn6/11$^{fl/fl}$**
PD1 MFI	3560	4760
	3218	4034
	3011	4278

Fig 3 F - SubG1

	Ptpn6/11$^{fl/fl}$	GzmBcre Ptpn6/11$^{fl/fl}$
Day 4	7.01	7.53
	6.44	8.57
	6.82	10.3
Day 5	5.66	8.26
	5.18	8.02
	6.01	7.38
Day 6	4.8	5.34
	4.24	5.67
	4.23	5.58

Fig 3 H - Altered proteins

	Ptpn6/11$^{fl/fl}$	GzmBcre Ptpn6/11$^{fl/fl}$
Tnfrsf1b	8950	3225
	5768	1389
	10712	1955
Mcl-1	20164	5786
	16620	7596
	13812	5954
