# Supplementary material for: Concomitant deletion of Ptpn6 and Ptpn11 in T cells fails to improve anticancer responses
Source: EMBO Rep. 2022 Oct 4;23(11):e55399. doi: 10.15252/embr.202255399 (PMC9638855; doi:10.15252/embr.202255399)
Supplement: Supplementary file 8 [file EMBR-23-e55399-s002.pdf]

EMBO reports

Concomitant deletion of Ptpn6 and Ptpn11 in T cells fails to improve anticancer responses

Pedro Ventura, Milica Gakovic, Berenice Fischer, Laura Spinelli, Giorgia Rota, Shalini Pathak, Hanif Khameneh, Alessandro Zenobi, Sarah Thomson, Walter Birchmeier, Doreen A. Cantrell, and Greta Guarda
DOI: 10.15252/embr.202255399

Corresponding author(s): Greta Guarda (Greta.Guarda@irb.usi.ch) , Doreen A. Cantrell (d.a.cantrell@dundee.ac.uk)

Review Timeline:

Submission Date:	16th May 22
Editorial Decision:	20th Jun 22
Revision Received:	8th Aug 22
Editorial Decision:	25th Aug 22
Revision Received:	5th Sep 22
Accepted:	8th Sep 22

Editor: Achim Breiling

Transaction Report:

(Note: With the exception of the correction of typographical or spelling errors that could be a source of ambiguity, letters and reports are not edited. Depending on transfer agreements, referee reports obtained elsewhere may or may not be included in this compilation. Referee reports are anonymous unless the Referee chooses to sign their reports.)

1st Editorial Decision **20th Jun 2022**

Dear Prof. Guarda,

Thank you for the submission of your research manuscript to EMBO reports. I have now received the reports from the three referees that were asked to evaluate your study, which can be found at the end of this email.

As you will see, the referees think that these findings are of interest. However, all referees have several comments, concerns, and suggestions, indicating that revision of the manuscript is necessary to allow publication of the study in EMBO reports. As the reports are below, and all their points need to be addressed, I will not detail them here.

Given the constructive referee comments, we would like to invite you to revise your manuscript with the understanding that all referee concerns must be addressed in the revised manuscript and in a detailed point-by-point response. Acceptance of your manuscript will depend on a positive outcome of a second round of review. It is EMBO reports policy to allow a single round of revision only and acceptance of the manuscript will therefore depend on the completeness of your responses included in the next, final version of the manuscript.

Revised manuscripts should be submitted within three months of a request for revision. Please contact me to discuss the revision (also by video chat) if you have questions or comments regarding the revision, or should you need additional time.

When submitting your revised manuscript, please also carefully review the instructions that follow below.

PLEASE NOTE THAT upon resubmission revised manuscripts are subjected to an initial quality control prior to exposition to re-review. Upon failure in the initial quality control, the manuscripts are sent back to the authors, which may lead to delays. Frequent reasons for such a failure are the lack of the data availability section (please see below) and the presence of statistics based on n=2 (the authors are then asked to present scatter plots or provide more data points).

When submitting your revised manuscript, we will require:

1) a .docx formatted version of the final manuscript text (including legends for main figures, EV figures and tables), but without the figures included. Figure legends should be compiled at the end of the manuscript text.

2) individual production quality figure files as .eps, .tif, .jpg (one file per figure), of main figures and EV figures. Please upload these as separate, individual files upon re-submission.

The Expanded View format, which will be displayed in the main HTML of the paper in a collapsible format, has replaced the Supplementary information. You can submit up to 5 images as Expanded View. Please follow the nomenclature Figure EV1, Figure EV2 etc. The figure legend for these should be included in the main manuscript document file in a section called Expanded View Figure Legends after the main Figure Legends section. Additional Supplementary material should be supplied as a single pdf file labeled Appendix. The Appendix should have page numbers and needs to include a table of content on the first page (with page numbers) and legends for all content. Please follow the nomenclature Appendix Figure Sx, Appendix Table Sx etc. throughout the text, and also label the figures and tables according to this nomenclature.

For more details, please refer to our guide to authors:
http://www.embopress.org/page/journal/14693178/authorguide#manuscriptpreparation

Please consult our guide for figure preparation:
http://wol-prod-cdn.literatumonline.com/pb-assets/embo-site/EMBOPress_Figure_Guidelines_061115-1561436025777.pdf

See also the guidelines for figure legend preparation:
https://www.embopress.org/page/journal/14693178/authorguide#figureformat

3) a complete author checklist, which you can download from our author guidelines (https://www.embopress.org/page/journal/14693178/authorguide). Please insert page numbers in the checklist to indicate where the requested information can be found in the manuscript. The completed author checklist will also be part of the RPF.

Please also follow our guidelines for the use of living organisms, and the respective reporting guidelines:
http://www.embopress.org/page/journal/14693178/authorguide#livingorganisms

4) that primary datasets produced in this study (e.g. RNA-seq, ChIP-seq, structural and array data) are deposited in an appropriate public database. If no primary datasets have been deposited, please also state this in a dedicated section (e.g. 'No primary datasets have been generated and deposited'), see below.

See also: http://embor.embopress.org/authorguide#datadeposition

Please remember to provide a reviewer password if the datasets are not yet public.

The accession numbers and database should be listed in a formal "Data Availability" section (placed after Materials & Methods) that follows the model below. This is now mandatory (like the COI statement). Please note that the Data Availability Section is restricted to new primary data that are part of this study. This section is mandatory. As indicated above, if no primary datasets have been deposited, please state this in this section

Data availability

The datasets produced in this study are available in the following databases:

- RNA-Seq data: Gene Expression Omnibus GSE46843 (https://www.ncbi.nlm.nih.gov/geo/query/acc.cgi?acc=GSE46843)
- [data type]: [name of the resource] [accession number/identifier/doi] ([URL or identifiers.org/DATABASE:ACCESSION])

*** Note - All links should resolve to a page where the data can be accessed. ***

Moreover, I have these editorial requests:

6) We strongly encourage the publication of original source data with the aim of making primary data more accessible and transparent to the reader. The source data will be published in a separate source data file online along with the accepted manuscript and will be linked to the relevant figure. If you would like to use this opportunity, please submit the source data (for example scans of entire gels or blots, data points of graphs in an excel sheet, additional images, etc.) of your key experiments together with the revised manuscript. If you want to provide source data, please include size markers for scans of entire gels, label the scans with figure and panel number, and send one PDF file per figure.

7) Our journal encourages inclusion of *data citations in the reference list* to directly cite datasets that were re-used and obtained from public databases. Data citations in the article text are distinct from normal bibliographical citations and should directly link to the database records from which the data can be accessed. In the main text, data citations are formatted as follows: "Data ref: Smith et al, 2001" or "Data ref: NCBI Sequence Read Archive PRJNA342805, 2017". In the Reference list, data citations must be labeled with "[DATASET]". A data reference must provide the database name, accession number/identifiers and a resolvable link to the landing page from which the data can be accessed at the end of the reference. Further instructions are available at: http://www.embopress.org/page/journal/14693178/authorguide#referencesformat

8) Regarding data quantification and statistics, please make sure that the number "n" for how many independent experiments were performed, their nature (biological versus technical replicates), the bars and error bars (e.g. SEM, SD) and the test used to calculate p-values is indicated in the respective figure legends (also for potential EV figures and all those in the final Appendix). Please also check that all the p-values are explained in the legend, and that these fit to those shown in the figure. Please provide statistical testing where applicable. Please avoid the phrase 'independent experiment', but clearly state if these were biological or technical replicates. See also:
http://www.embopress.org/page/journal/14693178/authorguide#statisticalanalysis

9) Please also note our reference format:
http://www.embopress.org/page/journal/14693178/authorguide#referencesformat

10) We updated our journal's competing interests policy in January 2022 and request authors to consider both actual and perceived competing interests. Please review the policy https://www.embopress.org/competing-interests and update your competing interests if necessary. Please name this section 'Disclosure and Competing Interests Statement' and put it after the Acknowledgements section.

11) Please add up to five keywords to the title page.

I look forward to seeing a revised version of your manuscript when it is ready. Please let me know if you have questions or comments regarding the revision.

Please use this link to submit your revision: https://embor.msubmit.net/cgi-bin/main.plex

Yours sincerely,

Achim Breiling
Senior Editor
EMBO Reports

Referee #1:

In this concise paper, Venture et al. studied the effects of SHP1 or SHP1/2 deletion in polyclonal T cells rather than SHP1 knockdown in transgenic T cells as in previous studies (Snook et al., Cancer Immunology Res, 2020). In this polyclonal setting, the authors found that SHP1 deletion or SHP1/SHP2 double deletion, driven by CD4-Cre, had little effect on T cell mediated control of the immunogenic MC38 tumor model. However, in the presence of PD1 blockade, SHP1/2-deletion impaired the anti-tumor activity of anti-PD1, correlated with a decreased of tumor infiltrating CD8+ T cells. Moreover, acute deletion of SHP1/2 in peripheral CD8 T cells, driven by granzyme-B-Cre, also failed to promote the anti-tumor response. They then showed using in vitro cultures that SHP1/2-deficiency altered the proteome of CD8+ T cells, especially the apoptosis-related pathways. This work highlights the complexity of SHP1 and SHP2 in regulating anti-tumor immunity of T cells, and suggests caution against the use of SHP1/2 pharmacological inhibitors in immunotherapies, because these inhibitors would inevitably target SHP1/2 in polyclonal T cell populations, though this study have not explored the SHP1/2 deficiency in non-T-cells which should also be targeted by SHP1/2 pharmacological inhibitors. I feel that the findings are important to report and may interest both immunologists and cancer biologists, but the study is a bit preliminary at this stage. A main weakness of the work is its somewhat descriptive nature with little mechanism.

Major concerns:
1. This reviewer feels that it will be important to dissect the cell type specific functions of SHP1 and SHP2. In particular, how SHP1 and SHP1/2 deletion in CD8+ T cells affect the anti-tumor activity. Some findings in this paper contradict Snook et al., who tested SHP1 knockdown in CD8+ TCR-transgenic T cells. Is it possible for the authors to transfer SHP1 KO and SHP1/2 KO CD8+ T cells to their existing tumor model? This experiment should be doable with their well-established knockout systems, and I believe that this experiment may provide valuable insight that improve the mechanistic clarity of this work.
2. Fig. 1C, SHP1 KO from polyclonal T cells did not affect the survival of mice in the absence of anti-PD1 but impaired the tumor control in the presence of anti-PD1. This data is difficult to understand. It seems to suggest that when the PD1 pathway is blocked, SHP1 contributes to the anti-tumor activity of T cells (why?), but when PD1 pathway is present, the net contribution of SHP1 to T cell activity is minimal. Presumably, the presence of SHP1 should increase the threshold of TCR signaling, rendering T cells less likely to response to weak tumor antigens. In this model, SHP1 KO should promote the anti-tumor response. Was the paradoxical effect of SHP1 KO due to Tregs? How do SHP KO affect Treg numbers and functions?
3. This reviewer is curious about why the authors did not test SHP2 KO, together with SHP1 KO and double KO, especially given that they already generated the SHP2 KO mice in their previous work (Rota et al., Cell Rep, 2018). Testing SHP2 KO in the same tumor model would be valuable in comparing SHP1 and SHP2 functions and their potential redundancy.
4. Fig. 2, the granzyme-B-Cre system allowed the authors to specifically delete SHP1/2 in peripheral CD8+ T cells, but the anti-PD1 treatment condition was lacking. Does SHP1/2-deletion compromise anti-PD1 therapy in this setting?
5. A key implication of the SHP KO data in this paper is that SHP1/2 inhibitors would blunt the anti-PD1 response in a MC38 model. This seems to be testable using the existing SHP1/2 inhibitors, such as SHP099. Can the authors please provide some evidence along this line?
6. Given the discrepancies between this work and prior studies in transgenic models, the paper can be strengthened by confirming key findings in a different immunogenic model, if the authors have one at hand.

Minor concerns:
1. Order of presentation is confusing, Fig. 1C is mentioned before Fig. 1B in the main text.
2. The term Bgn and Wbm are confusing, should be better defined at first mention.
3. Fig. 1G, H focused on CD8+ T cells, was there a reason that the authors did not check the frequency of CD4+ T cells as in some other panels?

Referee #2:

Does this manuscript report a single key finding?

Yes, the authors describe in their publication that T-cell intrinsic deletion of SHP-1 and SHP-2 fails to improve anticancer responses.

Is the work of significance? // Is it of general interest to the molecular biology immunity?

Yes, the work presented by Ventura et al. is of significance and general interest to the molecular biology community, with additional special relevance to the fields of cancer immunology and molecular immunology.

The protein phosphatases SHP-1 and SHP-2 function as critical signal transduction regulators and are known to interact with key signaling molecules such as the PD-1 pathway. Targeting these two phosphatases may be a promising therapeutic strategy, either alone or in combination with other inhibitors such as PD-1. To date, most reports have focused on the direct cancer cell-related effects. To which extent SHP-1 and SHP-2 deletion in T cells might have a benefit for tumor control has not been

investigated. So far, only data from CD4 Cre ptpn 11 fl/fl (T cell specific SHP-2 deletion) are available (1, 2). In the present report the authors study the tumor control in mice with T cell specific deletion of either SHP-1 alone or of SHP-1 and SHP-2.

Such studies help to gauge the promise of SHP2 and SHP1 as drug targets, such as by allosteric inhibitors that are considered for cancer immunotherapy.

Major points:

1.) The authors report tumor volume data: do the tumors of wildtype and knockout mice grow at the same rate and do they reach the same size? The text states: "Our data showed that tumors grew equally in control versus CD4Cre Ptpn6 fl/fl mice". This information cannot be obtained from Fig. 1C showing only the survival curves. The same applies to the experiments performed with CD4 Cre Ptpn6/11 fl/fl mice. Tumor volume curves should be added to accompany all tumor survival curves.
2.) The authors conclude that in steady state, Gzmb Cre Ptpng/6-11 fl/fl mice do not reveal major alterations (Fig. EV2A). As shown for the CD4 Cre ptpn6/11 fl/fl mouse, the main differences in terms of total cell number are detectable in the lymph nodes only but not in spleen (Fig. EV1C). For the Gzmb Cre Ptpng6/11 fl/fl mice Fig. EV2 reports the values for the spleen only, whereas data from lymph nodes are missing. These data should be added to exclude a potential effect of Gzmb Cre Ptpng6/11 fl/fl on T cell populations in steady state.
3.) "In agreement, control of tumor growth is affected when T cells lack Shp-1/2, in the presence of absence of anti-PD-1 treatment". "The loss of Shp-1/2 also impairs the therapeutic effects of anti-PD-1 treatment." It should be stated that this effect on the efficiency of PD-1 therapy was only tested in model with sustained Shp-1/-2 deficiency. Alternatively, data should be included to show that the same holds true in the Gzmb Cre Ptpng6/11 fl/fl mouse.

Minor points that should be changed:

• CD4 T cells are known to contribute to tumor regression by diverse mechanism. The authors are using a CD4 Cre mouse that targets CD4 as well as CD8 T cells (as illustrated in Fig. 1 A), the tumor-infiltrating CD4 T cell response (and potential effects of Shp-1/-2 deficiency thereupon) is not studied. Do you suspect that similarly to what was observed in CD4 Cre Ptpn11fl/fl mice, the antitumoral CD8 T cell response would not be inherently different when CD4 T cells were depleted? Please discuss this and the contribution of CD4 T cell responses to tumor control.
• In contrast to CD4 Cre Ptpn6 fl/fl mice, CD4Cre Ptpn11fl/fl mice show normal anticancer responses and therapeutic effects upon PD-1 blockade (as shown in (2)). Please discuss this.
• Rework your axis labels, e.g. -> Fig. 1H "% of CD8 T cells" or Fig. 2D "% of PD-1+ / CD8+ YFP+ TILs». It is unclear what was measured. Please label as "x of y (%)" throughout.
• The numbers in the FACS plots are hard to see, please enlarge (e.g. Fig. 1D, Fig. 2C).
• Fig. 3A :The FACS plot comprise a lot of cell debris, please include the gate that was used for further analysis (such as defining the percentage of PD-1-expressing cells).
• It is unclear whether in graphs that don't report statistical analyses (e.g. Fig. EV2) testing has not been done or, alternatively, whether any potential differences were not statistically significant. Please specify as e.g. "n.s." in the latter case.
• Please re-phrase "our data indicating that blockade of both phosphatases fails to improve anticancer responses and translates into impaired PD-1 blockade in vivo is relevant and timely." The data presented in this study show solely that T cell specific deletion of SHP-1/SHP-2 fail to improve anticancer responses. This may suggest but does not formally test the possibility that the pharmacological blockade of these two phosphatases fails to improve anticancer responses.

References:
1. M. Zhao et al., SHP2 inhibition triggers anti-tumor immunity and synergizes with PD-1 blockade. Acta Pharm Sin B 9, 304-315 (2019).
2. G. Rota et al., Shp-2 Is Dispensable for Establishing T Cell Exhaustion and for PD-1 Signaling In Vivo. Cell Rep 23, 39-49 (2018).

Referee #3:

In this study, the authors investigate whether loss of SHP-1 and/or SHP-2 in T cells improves anti-tumor responses. Loss of SHP-1 not only fails to improve anti-tumor response but in fact counteracts any anti-PD-1-mediated effects. A comparable phenotype was observed when both SHP-1/SHP-2 were deleted using the same CD4-Cre system. While the loss of SHP-1/SHP-2 didn't seem to affect the overall T cell cytokine production, the number of CD8+ TIL T cells was greatly reduced upon SHP-1/SHP-2 deletion, both with and without anti-PD-1 treatment. Using a Granzyme B-Cre-based approach to delete SHP-1/SHP-2 in effector cells similarly failed to improve any anti-tumor response. In vitro culture of CD8+ T cell isolated from Granzyme B-Cre- SHP-1/SHP-2 floxed mice, showed a reduced expansion of SHP-1/SHP-2-deficient T cells over time. A proteomics approach identified substantial differences between mutant and control cells, indicating that the reduced expansion might be caused by impaired survival.
While this study is clinically relevant, especially in the context of the potential use of phosphatase inhibitors as part of anti-tumor

treatments, the data presented here are missing controls and leaving numerous critical questions unanswered, which substantially lowers the impact of the study.

The following questions need to be addressed to draw clear conclusions based on this study:
1. Is the observed increased cell death in the absence of the phosphatase a general survival defect or is it due to activation-induced cell death, which might be heightened due to the increased signaling downstream of the TCR in the absence of the PTPs.
2. Only one optimal non-physiological condition of anti-CD3/CD28 stimulation is being analyzed (Fig. 3). Considering that loss of SHP-1 leads to increased TCR signaling, the study should be repeated with a dose response including sub-optimal more physiological conditions to better mimic the in vivo anti-tumor scenario.
3. Most of the studies are done in the context of deleting both SHP-1 and SHP-2 and should include individual deletions to not only better compare them to previous studies, but also to identify unique pathways regulated by these PTPs. Moreover, the inhibitors supposed to be used in the clinic are ideally targeting only 1 of the PTPs.
4. The decrease of TILs shown in Figure 1 could have several reasons, lack of expansion, lack of migration, lack of survival. This should be addressed.

Point-by-point reply:

Reviewer #1
In this concise paper, Venture et al. studied the effects of SHP1 or SHP1/2 deletion in polyclonal T cells rather than SHP1 knockdown in transgenic T cells as in previous studies (Snook et al., Cancer Immunology Res, 2020). In this polyclonal setting, the authors found that SHP1 deletion or SHP1/SHP2 double deletion, driven by CD4-Cre, had little effect on T cell mediated control of the immunogenic MC38 tumor model. However, in the presence of PD1 blockade, SHP1/2-deletion impaired the anti-tumor activity of anti-PD1, correlated with a decreased of tumor infiltrating CD8+ T cells. Moreover, acute deletion of SHP1/2 in peripheral CD8 T cells, driven by granzyme-B-Cre, also failed to promote the anti-tumor response. They then showed using in vitro cultures that SHP1/2-deficiency altered the proteome of CD8+ T cells, especially the apoptosis-related pathways. This work highlights the complexity of SHP1 and SHP2 in regulating anti-tumor immunity of T cells, and suggests caution against the use of SHP1/2 pharmacological inhibitors in immunotherapies, because these inhibitors would inevitably target SHP1/2 in polyclonal T cell populations, though this study have not explored the SHP1/2 deficiency in non-T-cells which should also be targeted by SHP1/2 pharmacological inhibitors. I feel that the findings are important to report and may interest both immunologists and cancer biologists, but the study is a bit preliminary at this stage. A main weakness of the work is its somewhat descriptive nature with little mechanism.

We thank Reviewer 1 for the positive assessment of our work and have addressed the major concerns below.

Major concerns:
1. This reviewer feels that it will be important to dissect the cell type specific functions of SHP1 and SHP2. In particular, how SHP1 and SHP1/2 deletion in CD8+ T cells affect the anti-tumor activity.
In line with the suggestion of Reviewer 1 we used – among others – a GzmBcre strain to delete the phosphatases in activated $CD8^+$ T cells. These experiments, addressing our key question on whether the combined ablation of Shp-1 and Shp-2 reinvigorates antitumor responses, are important to report in this concise manuscript.

Some findings in this paper contradict Snook et al., who tested SHP1 knockdown in CD8+ TCR-transgenic T cells.
We understand the Reviewer's concern; we were expecting an improved antitumoral response by T cells lacking Shp-1/2 or Shp-1 based on the dampening activity of the latter on TCR signaling. However, independent results from the Cantrell and the Guarda laboratories proved the opposite. This is why we believe our data are important for the scientific community. There are however many reasons for the different outcomes of this and the study by Snook et al..
Snook et al. use interfering siRNA to block Shp-1 expression and achieve 70% knockdown where we have a total knockout. They also use adoptively transferred OT-1 transgenic T cells to target B16-F10 melanoma tumors that express altered peptide ligands of chicken ovalbumin. In contrast, we are looking at endogenous polyclonal T cell responses to MC38-derived tumour-associated antigens, a totally different model possibly better mirroring endogenous immune responses to human tumours.
The polyclonal TCR repertoire is shaped in the thymus and upon activation, which will not apply to the setting employed by Snook et al.. In fact, recent data show how Shp-1 deficiency promotes negative selection, decreasing mature thymocytes in a polyclonal population (Martinez et al., 2016). Not only the TCR repertoire of OT-1 transgenic T cells is not altered by Shp-1 deletion, but Shp-1 deletion is also less likely to affect negative selection of high affinity TCRs specific for non-self-antigens, as it is the case for the OT-1 TCR (Martinez et al., 2016, Schmitt et al., 2013). In support of an altered negative selection in our CD4cre Shp-1/2$^{fl/fl}$ mice, we added data (Figure EV1C) showing a decrease in frequency of single-positive CD4 and CD8 thymocytes. To circumvent thymic selection defects, the GzmBcre setting was used for the concomitant deletion of Shp-1 and Shp-2; also this approach did not lead to an improved antitumor response. Given the concomitant deletion of both phosphatases and the reasons mentioned above, this result is not in contradiction with the findings of Snook. et al. while it addresses the key question of this concise manuscript (Celis-Gutierrez et al., 2019).
Snook et al conclude that targeting Shp-1 expands the repertoire of T cells available to respond to conventional checkpoint blockade, leading to enhanced control of tumour growth. Our data show this is not true with another tumour model which relies on endogenous polyclonal $CD8^+$ T cell-mediated immune responses. The fact that two independent groups obtained these results highlights how targeting Shp-1 does not always have a good outcome for antitumour immune responses; the differences underlying these studies have been further highlighted in the results and in the discussion.

Is it possible for the authors to transfer SHP1 KO and SHP1/2 KO CD8+ T cells to their existing tumor model? This experiment should be doable with their well-established knockout systems, and I believe that this experiment may provide valuable insight that improve the mechanistic clarity of this work.
Concerning the proposal to transfer Shp-1- and Shp-1/2-deficient CD8$^+$ T cells to MC38-bearing mice; we do not feel this experiment will be informative since the T cells we are studying are polyclonal and not transgenic for a MC38-specific TCR. Hence, they will not accumulate in the MC38 tumor microenvironment and make the experiment uninformative. We do feel that we have mechanistic data in the paper, not least the proteomic analysis revealing the severe consequences of Shp-1/2 deletion and how it impacts the effector T cell phenotype.

2. Fig. 1C - 1D in the revised manuscript -**, SHP1 KO from polyclonal T cells did not affect the survival of mice in the absence of anti-PD1 but impaired the tumor control in the presence of anti-PD1. This data is difficult to understand. It seems to suggest that when the PD1 pathway is blocked, SHP1 contributes to the anti-tumor activity of T cells (why?), but when PD1 pathway is present, the net contribution of SHP1 to T cell activity is minimal.**
Upon PD-1 blockade, mice with Shp-1-deleted T cells still respond to the treatment but less as compared to control mice, revealing a partial defect in these cells' response to anti-PD-1 therapy (this is also visible from the tumor growth curves that we added to Figure 1C). If indeed the effectiveness of the CD8$^+$ T cell response is partially impaired by Shp-1 deletion, this is not likely to be revealed in isotype-treated MC38-bearing mice, where the contribution of CD8$^+$ T cells to MC38 control is minor, but rather upon anti-PD1 treatment, which amplifies the role of CD8$^+$ T cells (Jin et al., 2022).

Presumably, the presence of SHP1 should increase the threshold of TCR signaling, rendering T cells less likely to response to weak tumor antigens. In this model, SHP1 KO should promote the anti-tumor response.
As Shp-1 deficiency promotes negative selection, it is expected that the most affected thymocytes are the ones with highest signal strength, e.g. with reactivity for self-antigens (Schmitt et al., 2013, Martinez et al., 2016). This will in turn impair the response to endogenous tumor-associated antigens (Schmitt et al., 2013).

Was the paradoxical effect of SHP1 KO due to Tregs? How do SHP KO affect Treg numbers and functions?
The explanation above is not mutually exclusive with the suggestion by Reviewer 1, whereby Tregs might play a part. We analyzed Tregs and added data to Figure EV1A showing a mild increase in Tregs frequency but not in number in Cd4cre *Ptpn6*$^{fl/fl}$ mice. Similarly, Tregs in Cd4cre *Ptpn6/11*$^{fl/fl}$ mice are augmented in frequency but their numbers are not significantly altered (Figure EV1D). In line with the normal numbers of Tregs, data from a single experiment show unaltered Treg frequencies among MC38-infiltrating leukocytes, reason why we did not further pursue this aspect (see Figure below). While the use of inhibitors would likely affect the T cell – including the Treg – compartment similarly to what we observed in Cd4cre *Ptpn6/11*$^{fl/fl}$ mice, the data generated with GzmBcre deletion in effector CD8$^+$ T cells speak against a major role by Tregs to explain the ineffective antitumoral performances by Shp-1/2-deleted T cells.

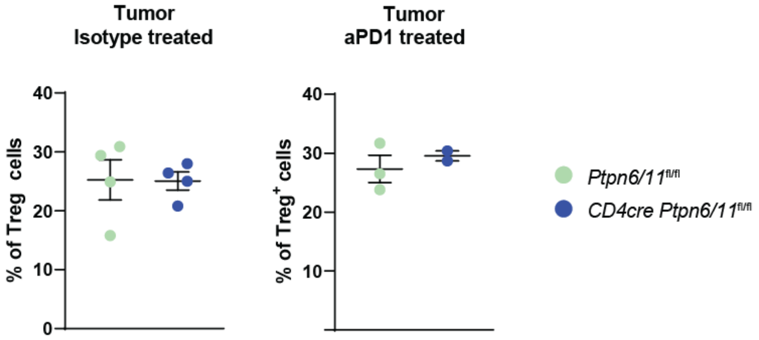

10-12 days following MC38 tumour inoculation and treatment with anti-PD-1 antibody or isotype control, mice of the indicated genotypes were sacrificed, and regulatory T cells analysed. Graphs depict frequency of Foxp3$^+$ T cells (gated among CD45$^+$ TCR$^+$ CD4$^+$) in the tumour. Results depict n=2-4 mice/group and Student's t test (unpaired, two-tailed) was used to compare differences between experimental groups.

3. This reviewer is curious about why the authors did not test SHP2 KO, together with SHP1 KO and double KO, especially given that they already generated the SHP2 KO mice in their previous work (Rota et al., Cell Rep, 2018). Testing SHP2 KO in the same tumor model would be valuable in comparing SHP1 and SHP2 functions and their potential redundancy.

As alluded to, the Guarda's laboratory – one of the two laboratories involved in this study – performed an extensive study of mice lacking Shp-2 in T cells including the same immunogenic cancer model (MC38) used here ((Rota et al., 2018) see Figure 5). For ethical reasons, we do not deem it acceptable to repeat the same animal studies, as they are not likely to provide new knowledge as compared to our previous results, which meantime were confirmed by others in similar settings (Quintana et al., 2020, Boussiotis et al., 2022). Nonetheless, previous results on Shp-2-deficient T cells by the Guarda group have been highlighted in the discussion.

4. Fig. 2, the granzyme-B-Cre system allowed the authors to specifically delete SHP1/2 in peripheral CD8+ T cells, but the anti-PD1 treatment condition was lacking. Does SHP1/2-deletion compromise anti-PD1 therapy in this setting?

We recognize the Reviewer's point and performed a set of experiments using GzmBcreER-T2 *Ptpn6/11*$^{fl/fl}$ mice (unfortunately, due to pressures caused by the COVID pandemic, we had the stop maintaining the GzmBcre *Ptpn6/11*$^{fl/fl}$ mice). In this genetic model, the Cre recombinase is fused with a modified form of the estrogen receptor (CreER-T2) that prevents the Cre ER-T2 fusion protein from accessing the nucleus, unless in the presence of tamoxifen (Bannard et al., 2009). Being placed under the Granzyme B (GzmB) promotor, also this construct allows to specifically target the cytotoxic cells. Despite we decided not to include these data in the manuscript because of the suboptimal deletion (only 10% of the tumor-infiltrating CD8$^+$ T cells, as compared to the 50% reached with the model used in Figure 2), we still find the data suggestive of an incomplete response to anti-PD1 treatment by T cells acutely deleted for Shp-1/2, with a *p* value very close to significance.

In spite of this, for what concerns the manuscript data, restricting Shp-1/2 deletion to CTLs shall significantly limit any confounding effect on T cell homeostasis, enabling to uncover the potential role of these phosphatases in exhausted T cells' reinvigoration even in the absence of anti-PD-1 treatment.

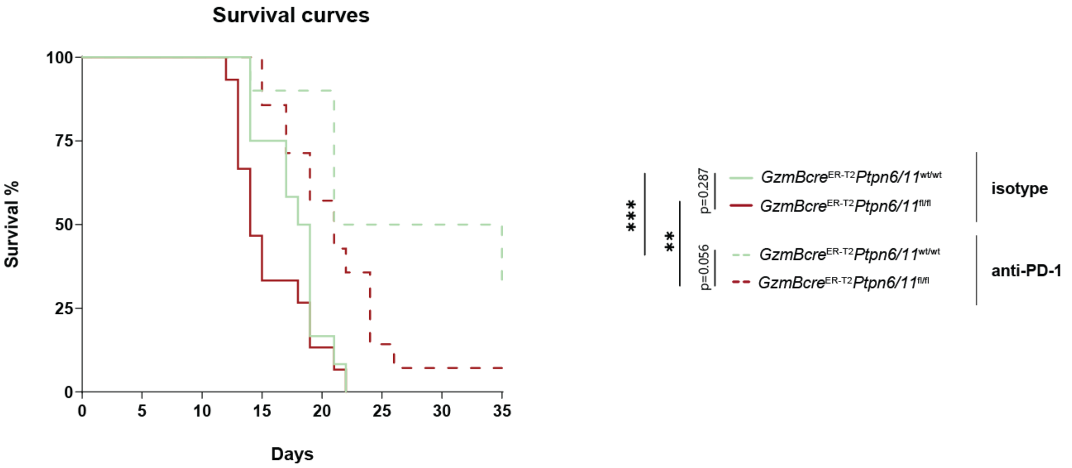

Survival curves of *GzmBcreER-T2 Ptpn6/11*$^{wt/wt}$ and *GzmBcreER-T2 Ptpn6/11*$^{fl/fl}$ mice upon subcutaneous MC38 engraftment. Mice of the indicated genotype were treated with either isotype control or anti-PD-1 antibody. Results depict n=10-15 mice/group and are a pool of three independent experiments. For survival statistics, comparisons were done in GraphPad PRISM using the log-rank (Mantel-Cox) test.

5. A key implication of the SHP KO data in this paper is that SHP1/2 inhibitors would blunt the anti-PD1 response in a MC38 model. This seems to be testable using the existing SHP1/2 inhibitors, such as SHP099. Can the authors please provide some evidence along this line?

These inhibitors are in clinical trials, mostly to target cancerous cells directly, so the results of how beneficial they are will soon be known (Yi et al., 2002, Mullard, 2018). So far, published data indicate that Shp1/2 blockade with sodium stibogluconate administered in combination with IFN alpha did not induce objective disease regression in a Phase I study (Yi et al., 2011); recent preclinical data suggested however that sodium stibogluconate might act on neutrophil-mediated ADCC when administered with cancer-specific therapeutic antibodies (van Rees et al., 2022). Preclinical data for Shp-2 blockade show effects on cancer cells, myeloid, and lymphoid subsets (Mullard, 2018, Chen et al., 2016, Wang et al., 2021, Quintana et al., 2020). With respect to immune cells, emerging data indicate that the major role for Shp-2 is in myeloid cells, and not in T cells, compatibly with previous data by us (Boussiotis et al., 2022, Rota et al., 2018).

Thus, as the use of inhibitors will act on every cell, including tumor and myeloid cells in many cases, we deem this approach not amenable to investigate the role of Shp-1/2 in T cells, which is our goal. We conveyed the message more clearly in the modified discussion, stating that inhibitors might have beneficial effects on multiple cancers and that our data are willingly limited to the role of Shp-1/2 in T cells.

6. Given the discrepancies between this work and prior studies in transgenic models, the paper can be strengthened by confirming key findings in a different immunogenic model, if the authors have one at hand.
For the reasons mentioned in the answer to point 1, we do not see a contradiction between our study and prior studies with transgenic models. It is also important to underline that our scope is to analyze the response of Shp-1/2 double-deficient T cells, given the suggestion that redundancy with Shp-1 could explain the lack of T cell reinvigoration observed in the absence of Shp-2 (Celis-Gutierrez et al., 2019). Shp-1-deficient T cells have been studied for selected aspects to enrich the study and allow a direct evaluation of the effects of each phosphatase. The model we chose, the MC38 colon adenocarcinoma, is an established model for preclinical studies on checkpoint inhibitors (Juneja et al., 2017). In addition, unfortunately, due to pressures caused by the COVID pandemic and by a relocation of the animal facility, we had the stop maintaining the relevant SHP1/2 mouse colonies, precluding us the possibility to perform this experiment.

Minor concerns:
1. Order of presentation is confusing, Fig. 1C is mentioned before Fig. 1B in the main text.
We apologize for any confusion and have amended Figure and text.

2. The term Bgn and Wbm are confusing, should be better defined at first mention.
This has been amended at first mention.

3. Fig. 1G, H focused on CD8+ T cells, was there a reason that the authors did not check the frequency of CD4+ T cells as in some other panels?
We understand the importance of this point and analyzed the frequency and cytokine expression of tumor-infiltrating $CD4^+$ T cells, without however observing significant differences. These data have been included in Figure EV1H, I.

Reviewer #2

Does this manuscript report a single key finding?

Yes, the authors describe in their publication that T-cell intrinsic deletion of SHP-1 and SHP-2 fails to improve anticancer responses.

Is the work of significance? // Is it of general interest to the molecular biology immunity?

Yes, the work presented by Ventura et al. is of significance and general interest to the molecular biology community, with additional special relevance to the fields of cancer immunology and molecular immunology.

The protein phosphatases SHP-1 and SHP-2 function as critical signal transduction regulators and are known to interact with key signaling molecules such as the PD-1 pathway. Targeting these two phosphatases may be a promising therapeutic strategy, either alone or in combination with other inhibitors such as PD-1. To date, most reports have focused on the direct cancer cell-related effects. To which extent SHP-1 and SHP-2 deletion in T cells might have a benefit for tumor control has not been investigated. So far, only data from CD4 Cre ptpn 11 fl/fl (T cell specific SHP-2 deletion) are available (1, 2). In the present report the authors study the tumor control in mice with T cell specific deletion of either SHP-1 alone or of SHP-1 and SHP-2.

Such studies help to gauge the promise of SHP2 and SHP1 as drug targets, such as by allosteric inhibitors that are considered for cancer immunotherapy.

We would like to thank Reviewer 2 for their many positive comments on the manuscript and the recognition of the significance of our work for the cancer immunotherapy community. Major concerns are addressed below.

Major points:
1.) The authors report tumor volume data: do the tumors of wildtype and knockout mice grow at the same rate and do they reach the same size? The text states: "Our data showed that tumors grew equally in control versus CD4Cre Ptpn6 fl/fl mice". This information cannot be obtained from Fig. 1C - 1D in the revised manuscript - **showing only the survival curves. The same applies to the experiments performed with CD4 Cre Ptpn6/11 fl/fl mice. Tumor volume curves should be added to accompany all tumor survival curves.**
We understand the Reviewer’s concern and included the requested data in Figure 1C, 1G and EV2E.

2.) The authors conclude that in steady state, Gzmb Cre Ptpng/6-11 fl/fl mice do not reveal major alterations (Fig. EV2A). As shown for the CD4 Cre ptpn6/11 fl/fl mouse, the main differences in terms of total cell number are detectable in the lymph nodes only but not in spleen (Fig. EV1C). For the Gzmb Cre Ptpng6/11 fl/fl mice Fig. EV2 reports the values for the spleen only, whereas data from lymph nodes are missing. These data should be added to exclude a potential effect of Gzmb Cre Ptpng6/11 fl/fl on T cell populations in steady state.
We added data relative to lymph nodes to Figure EV2B. These show that the number of T cells and their levels of CD44 are normal in *GzmBcre Ptpn6/11*$^{fl/fl}$ mice.

3.) "In agreement, control of tumor growth is affected when T cells lack Shp-1/2, in the presence of absence of anti-PD-1 treatment". "The loss of Shp-1/2 also impairs the therapeutic effects of anti-PD-1 treatment." It should be stated that this effect on the efficiency of PD-1 therapy was only tested in model with sustained Shp-1/-2 deficiency. Alternatively, data should be included to show that the same holds true in the Gzmb Cre Ptpng6/11 fl/fl mouse.
We amended the text accordingly in the abstract and the discussion; for the answer to the last query, please refer to Referee 1 point 4.

Minor points that should be changed:

• CD4 T cells are known to contribute to tumor regression by diverse mechanism. The authors are using a CD4 Cre mouse that targets CD4 as well as CD8 T cells (as illustrated in Fig. 1 A), the tumor-infiltrating CD4 T cell response (and potential effects of Shp-1/-2 deficiency thereupon) is not studied. Do you suspect that similarly to what was observed in CD4 Cre Ptpn11fl/fl mice, the antitumoral CD8 T cell response would not be inherently different when CD4 T cells were depleted? Please discuss this and the contribution of CD4 T cell responses to tumor control.
We have analyzed the frequency and cytokine expression of tumor-infiltrating CD4$^+$ T cells and no significant differences were observed. These data have been included in Figure EV1H and I. Further, we added data on Tregs that are discussed in the last part of the answer to point 2 of Reviewer 1. While we cannot rule out a contribution of CD4$^+$ T cells to the dysfunctional antitumor responses in *CD4cre Ptpn6/11*$^{fl/fl}$ mice, our data generated with *Gzmbcre Ptpn6/11*$^{fl/fl}$ mice are not supporting a key role for CD4$^+$ T cells; these results have been now discussed in the manuscript, at the end of the second result section.

• In contrast to CD4Cre Ptpn6 fl/fl mice, CD4Cre Ptpn11fl/fl mice show normal anticancer responses and therapeutic effects upon PD-1 blockade (as shown in (2)). Please discuss this.
Despite their partial homology, the main roles reported for these phosphatases are distinct, with Shp-1 being involved in the regulation of antigen receptor signaling by targeting for example LCK and ZAP70 (Gaud et al., 2018) and Shp-2 being best known for its functional role in the activation of ERK downstream of multiple growth factor and signaling receptors (Niogret et al., 2019a). This has been more clearly conveyed in the introduction. In our view, the divergent effects of Shp-1 and Shp-2 deletion on T cells are thus not surprising and we raised this point in the discussion.

• Rework your axis labels, e.g. -> Fig. 1H "% of CD8 T cells" or Fig. 2D "% of PD-1+ / CD8+ YFP+ TILs». It is unclear what was measured. Please label as "x of y (%)" throughout.
We amended the Figures as suggested.

• The numbers in the FACS plots are hard to see, please enlarge (e.g. Fig. 1D, Fig. 2C).
We amended the Figures accordingly.

• Fig. 3A: The FACS plot comprise a lot of cell debris, please include the gate that was used for further analysis (such as defining the percentage of PD-1-expressing cells).
We thank the Referee for raising this point; we amended Figure 3A as suggested and added details on the gating in the Figure legend.

• It is unclear whether in graphs that don't report statistical analyses (e.g. Fig. EV2) testing has not been done or, alternatively, whether any potential differences were not statistically significant. Please specify as e.g. "n.s." in the latter case.
We amended the Figures accordingly.

• Please re-phrase "our data indicating that blockade of both phosphatases fails to improve anticancer responses and translates into impaired PD-1 blockade in vivo is relevant and timely." The data presented in this study show solely that T cell specific deletion of SHP-1/SHP-2 fail to improve anticancer responses.

This may suggest but does not formally test the possibility that the pharmacological blockade of these two phosphatases fails to improve anticancer responses.
We agree that we can only infer what inhibitor-mediate blockade might do in T cells; we therefore amended the text as requested.

Reviewer #3

In this study, the authors investigate whether loss of SHP-1 and/or SHP-2 in T cells improves anti-tumor responses. Loss of SHP-1 not only fails to improve anti-tumor response but in fact counteracts any anti-PD-1-mediated effects. A comparable phenotype was observed when both SHP-1/SHP-2 were deleted using the same CD4-Cre system. While the loss of SHP-1/SHP-2 didn't seem to affect the overall T cell cytokine production, the number of CD8+ TIL T cells was greatly reduced upon SHP-1/SHP-2 deletion, both with and without anti-PD-1 treatment. Using a Granzyme B-Cre-based approach to delete SHP-1/SHP-2 in effector cells similarly failed to improve any anti-tumor response. In vitro culture of CD8+ T cell isolated from Granzyme B-Cre- SHP-1/SHP-2 floxed mice, showed a reduced expansion of SHP-1/SHP-2-deficient T cells over time. A proteomics approach identified substantial differences between mutant and control cells, indicating that the reduced expansion might be caused by impaired survival.
While this study is clinically relevant, especially in the context of the potential use of phosphatase inhibitors as part of anti-tumor treatments, the data presented here are missing controls and leaving numerous critical questions unanswered, which substantially lowers the impact of the study.

We are grateful to this Referee for acknowledging the relevance of our study and believe that our responses below will alleviate any concerns about missing controls and feel that we can address some of the critical questions raised.

1. Is the observed increased cell death in the absence of the phosphatase a general survival defect or is it due to activation-induced cell death, which might be heightened due to the increased signaling downstream of the TCR in the absence of the PTPs.
This is a very good question. To address this point, we plated Shp-1/2 null and control cytotoxic T lymphocytes for 4h in untreated conditions, IL-2, or restimulated via their TCR with anti-CD3 antibody. Thereafter, total viable cell number was measured. Even if the partial convergence of the IL-2- and the TCR-mediated signaling cascades makes it difficult to fully uncouple these mechanisms, the data below show that TCR restimulation induces more cell death in Shp-1/2 null CTLs than in control CTLs and this effect is stronger than the one observed in IL-2-maintained or in non-treated cells. Data below are compatible with a general survival defect and an additional AICD problem, which might be relevant *in vivo*, where it cannot be easily tested – to this effect we added one sentence to the discussion acknowledging the possible contribution of AICD to the defective anticancer responses by Shp1/2 null T cells.

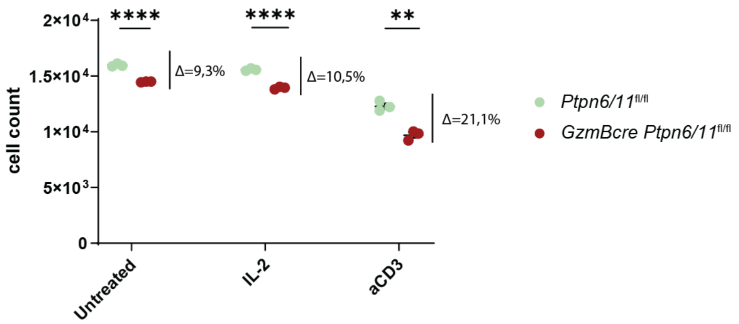

CTLs were generated from splenocytes of *GzmBcre Ptpn6/11*$^{fl/fl}$ and *Ptpn6/11*$^{fl/fl}$ mice by anti-CD3, anti-CD28, IL-2 and IL-12 stimulation for 48h, and then maintained in IL-2. On day 6, CTLs were collected, counted, and seeded in equal numbers on a plate coated with 1 ug/mL of anti-CD3 for 4h without the presence of cytokines. As controls, cells were plated on uncoated wells either without addition of cytokines or with IL-2 for 4h. Graph depicts numbers of CTLs counted after 4h in the indicated genotypes, percentage difference between Shp-1/2-deficient CTLs and their control counterparts are indicated in the graph. Results represent n=3 biological replicates, each based on the average value of n=2 technical replicates. Student's t test (unpaired, two-tailed) was used to compare differences between experimental groups.

However, as in the experiment presented in Figure 3C, phosphatase deletion happens upon expression of Granzyme B and indeed CTLs derived from *GzmBcre Ptpn6/11*$^{fl/fl}$ mice do not die more than their control counterparts up to 4 days after activation, we believe that the increased cell death shown in Figure 3C is independent from AICD.

2. Only one optimal non-physiological condition of anti-CD3/CD28 stimulation is being analyzed (Fig. 3). Considering that loss of SHP-1 leads to increased TCR signaling, the study should be repeated with a dose response including sub-optimal more physiological conditions to better mimic the in vivo anti-tumor scenario.

We understand the Reviewer's concern; unfortunately, due to pressures caused by the COVID pandemic, we had to stop maintaining the GzmBcre *Ptpn6/11*$^{fl/fl}$ strain and is therefore impossible for us to perform additional experiments. Notably, as in the experiments presented in Figure 3 phosphatase deletion happens upon expression of Granzyme B, it is unlikely that the initial TCR stimulation is largely affected by the absence of Shp1/2, as Shp-1 and Shp-2 are still present when the initial stimulation occurs.

Instead, a previous publication explored this important point and demonstrated that CD44-low polyclonal Shp-1-deficient $CD4^+$ and $CD8^+$ T cells did not respond better to sub-optimal anti-CD3/CD28 stimulation (Johnson et al., 2013). While this – as our – experimental set-up *in vitro* has the limitation of bypassing TCR specificity using anti-CD3 as a polyclonal stimulus, it is important to remember that our study investigates the antitumoral response *in vivo*. Data shown in Figure 1, Figure 2, and EV Figure 1 demonstrate that absence of Shp-1 or of Shp-1/2 in a polyclonal population is not leading to an improved response to suboptimal tumor antigens in physiological setting.

3. Most of the studies are done in the context of deleting both SHP-1 and SHP-2 and should include individual deletions to not only better compare them to previous studies, but also to identify unique pathways regulated by these PTPs. Moreover, the inhibitors supposed to be used in the clinic are ideally targeting only 1 of the PTPs.

We complemented the *in vivo* data using mice lacking Shp-1 in T cells (Figure 1C, D and Figure EV 1A, B). Moreover, the Guarda's laboratory – one of the two laboratories involved in this study – performed an extensive study of mice lacking Shp-2 in T cells including on the anti-MC38 T cell response ((Rota et al., 2018) see Figure 5). So, with respect to the key MC38 *in vivo* experiments, we provided data to compare the effects in T cells of each phosphatase.

If Reviewer 3 is instead referring to the *in vitro* studies, we would like to add that this is the object of an independent ongoing study on the signaling cascades regulated by Shp-2 in T cells. This bases on previous studies from the Guarda's laboratory and former work by others (Niogret et al., 2019a, Niogret et al., 2019b); given the complexity of the question, this will be object of a future manuscript and is therefore going beyond the scope of this study, which addresses in a short format if concomitant deletion of Shp-1 and Shp-2 in T cells reinvigorates anticancer responses.

Concerning the inhibitors; it is important to stress that sodium stibogluconate is currently used in clinical trials and blocks both phosphatases (see for instance the trials NCT00629200, NCT00498979, NCT04906031). Further, after the widely accepted role of Shp-2 in T cells' exhaustion has been challenged (Rota et al., 2018, Quintana et al., 2020, Boussiotis et al., 2022), the question addressed in this new work stems from the proposal that redundancy of Shp-1 and Shp-2 underlies the lack T cell reinvigoration observed in the absence of Shp-2 (Celis-Gutierrez et al., 2019). From here, our focus on studying the effects of the loss of both phosphatases's activity in T cells.

4. The decrease of TILs shown in Figure 1 could have several reasons, lack of expansion, lack of migration, lack of survival. This should be addressed.

This is an excellent point. However, it is not possible to think of an experimental way to disentangle lack of expansion of anti-MC38 T cells, from lack of migration, and lack of survival. One problem is that *in vivo*, there is very rapid clearance of dying cells by phagocytes. The second hurdle is that the T cells we are studying are not transgenic for MC38-specific TCR and are thus difficult to identify and will not accumulate in the tumor microenvironment upon transfer.

We thus addressed these aspects *in vitro*, where we observe no differences in cell cycle (EV Figure 3C), but a difference in survival (Figure 3C). In summary these are excellent suggestions, but it is not possible to think of an experimental way to resolve them. We have modified the discussion to acknowledge these limitations.

References

BANNARD, O., KRAMAN, M. & FEARON, D. T. 2009. Secondary replicative function of CD8+ T cells that had developed an effector phenotype. *Science,* 323**,** 505-9.

BOUSSIOTIS, V., CHRISTOFIDES, A., KATOPODI, X.-L., CAO, C., AKSOYLAR, H.-I., MAHMOUD, M., STRAUSS, L., TIJARO-OVALLE, N., PAL, R., BOON, L., ASARA, J., VLACHOS, I. & PATSOUKIS, N. 2022. SHP-2 and canonical PD-1: SHP-2 axis regulate myeloid cell differentiation, anti-tumor responses and innate immune memory. Research Square.

CELIS-GUTIERREZ, J., BLATTMANN, P., ZHAI, Y., JARMUZYNSKI, N., RUMINSKI, K., GREGOIRE, C., OUNOUGHENE, Y., FIORE, F., AEBERSOLD, R., RONCAGALLI, R., GSTAIGER, M. & MALISSEN, B. 2019. Quantitative Interactomics in Primary T Cells Provides a Rationale for Concomitant PD-1 and BTLA Coinhibitor Blockade in Cancer Immunotherapy. *Cell Rep,* 27**,** 3315-3330 e7.

CHEN, Y. P., LAMARCHE, M. J., CHAN, H. M., FEKKES, P., GARCIA-FORTANET, J., ACKER, M. G., ANTONAKOS, B., CHEN, C. H., CHEN, Z., COOKE, V. G., DOBSON, J. R., DENG, Z., FEI, F., FIRESTONE, B., FODOR, M., FRIDRICH, C., GAO, H., GRUNENFELDER, D., HAO, H. X., JACOB, J., HO, S., HSIAO, K., KANG, Z. B., KARKI, R., KATO, M., LARROW, J., LA BONTE, L. R., LENOIR, F., LIU, G., LIU, S., MAJUMDAR, D., MEYER, M. J., PALERMO, M., PEREZ, L., PU, M., PRICE, E., QUINN, C., SHAKYA, S., SHULTZ, M. D., SLISZ, J., VENKATESAN, K., WANG, P., WARMUTH, M., WILLIAMS, S., YANG, G., YUAN, J., ZHANG, J. H., ZHU, P., RAMSEY, T., KEEN, N. J., SELLERS, W. R., STAMS, T. & FORTIN, P. D. 2016. Allosteric inhibition of SHP2 phosphatase inhibits cancers driven by receptor tyrosine kinases. *Nature*.

GAUD, G., LESOURNE, R. & LOVE, P. E. 2018. Regulatory mechanisms in T cell receptor signalling. *Nat Rev Immunol,* 18**,** 485-497.

JIN, Y., AN, X., MAO, B., SUN, R., KUMARI, R., CHEN, X., SHAN, Y., ZANG, M., XU, L., MUNTEL, J., BEELER, K., BRUDERER, R., REITER, L., GUO, S., ZHOU, D., LI, Q. X. & OUYANG, X. 2022. Different syngeneic tumors show distinctive intrinsic tumor-immunity and mechanisms of actions (MOA) of anti-PD-1 treatment. *Sci Rep,* 12**,** 3278.

JOHNSON, D. J., PAO, L. I., DHANJI, S., MURAKAMI, K., OHASHI, P. S. & NEEL, B. G. 2013. Shp1 regulates T cell homeostasis by limiting IL-4 signals. *J Exp Med,* 210**,** 1419-31.

JUNEJA, V. R., MCGUIRE, K. A., MANGUSO, R. T., LAFLEUR, M. W., COLLINS, N., HAINING, W. N., FREEMAN, G. J. & SHARPE, A. H. 2017. PD-L1 on tumor cells is sufficient for immune evasion in immunogenic tumors and inhibits CD8 T cell cytotoxicity. *J Exp Med,* 214**,** 895-904.

MARTINEZ, R. J., MORRIS, A. B., NEELD, D. K. & EVAVOLD, B. D. 2016. Targeted loss of SHP1 in murine thymocytes dampens TCR signaling late in selection. *Eur J Immunol,* 46**,** 2103-10.

MULLARD, A. 2018. Phosphatases start shedding their stigma of undruggability. *Nat Rev Drug Discov,* 17**,** 847-849.

NIOGRET, C., BIRCHMEIER, W. & GUARDA, G. 2019a. SHP-2 in Lymphocytes' Cytokine and Inhibitory Receptor Signaling. *Front Immunol,* 10**,** 2468.

NIOGRET, C., MIAH, S. M. S., ROTA, G., FONTA, N. P., WANG, H., HELD, W., BIRCHMEIER, W., SEXL, V., YANG, W., VIVIER, E., HO, P. C., BROSSAY, L. & GUARDA, G. 2019b. Shp-2 is critical for ERK and metabolic engagement downstream of IL-15 receptor in NK cells. *Nat Commun,* 10**,** 1444.

QUINTANA, E., SCHULZE, C. J., MYERS, D. R., CHOY, T. J., MORDEC, K., WILDES, D., SHIFRIN, N. T., BELWAFA, A., KOLTUN, E. S., GILL, A. L., SINGH, M., KELSEY, S., GOLDSMITH, M. A., NICHOLS, R. & SMITH, J. A. M. 2020. Allosteric Inhibition of SHP2 Stimulates Antitumor Immunity by Transforming the Immunosuppressive Environment. *Cancer Res,* 80**,** 2889-2902.

ROTA, G., NIOGRET, C., DANG, A. T., BARROS, C. R., FONTA, N. P., ALFEI, F., MORGADO, L., ZEHN, D., BIRCHMEIER, W., VIVIER, E. & GUARDA, G. 2018. Shp-2 Is Dispensable for Establishing T Cell Exhaustion and for PD-1 Signaling In Vivo. *Cell Rep,* 23**,** 39-49.

SCHMITT, T. M., AGGEN, D. H., STROMNES, I. M., DOSSETT, M. L., RICHMAN, S. A., KRANZ, D. M. & GREENBERG, P. D. 2013. Enhanced-affinity murine T-cell receptors for tumor/self-antigens can be safe in gene therapy despite surpassing the threshold for thymic selection. *Blood,* 122**,** 348-56.
VAN REES, D. J., BRINKHAUS, M., KLEIN, B., VERKUIJLEN, P., TOOL, A. T. J., SCHORNAGEL, K., TREFFERS, L. W., VAN HOUDT, M., KATER, A. P., VIDARSSON, G., GENNERY, A. R., KUIJPERS, T. W., VAN BRUGGEN, R., MATLUNG, H. L. & VAN DEN BERG, T. K. 2022. Sodium stibogluconate and CD47-SIRPalpha blockade overcome resistance of anti-CD20-opsonized B cells to neutrophil killing. *Blood Adv,* 6**,** 2156-2166.
WANG, Y., MOHSENI, M., GRAUEL, A., DIEZ, J. E., GUAN, W., LIANG, S., CHOI, J. E., PU, M., CHEN, D., LASZEWSKI, T., SCHWARTZ, S., GU, J., MANSUR, L., BURKS, T., BRODEUR, L., VELAZQUEZ, R., KOVATS, S., PANT, B., BURUZULA, G., DENG, E., CHEN, J. T., SARI-SARRAF, F., DORNELAS, C., VARADARAJAN, M., YU, H., LIU, C., LIM, J., HAO, H. X., JIANG, X., MALAMAS, A., LAMARCHE, M. J., GEYER, F. C., MCLAUGHLIN, M., COSTA, C., WAGNER, J., RUDDY, D., JAYARAMAN, P., KIRKPATRICK, N. D., ZHANG, P., IARTCHOUK, O., AARDALEN, K., CREMASCO, V., DRANOFF, G., ENGELMAN, J. A., SILVER, S., WANG, H., HASTINGS, W. D. & GOLDONI, S. 2021. SHP2 blockade enhances anti-tumor immunity via tumor cell intrinsic and extrinsic mechanisms. *Sci Rep,* 11**,** 1399.
YI, T., ELSON, P., MITSUHASHI, M., JACOBS, B., HOLLOVARY, E., BUDD, T. G., SPIRO, T., TRIOZZI, P. & BORDEN, E. C. 2011. Phosphatase inhibitor, sodium stibogluconate, in combination with interferon (IFN) alpha 2b: phase I trials to identify pharmacodynamic and clinical effects. *Oncotarget,* 2**,** 1155-64.
YI, T., PATHAK, M. K., LINDNER, D. J., KETTERER, M. E., FARVER, C. & BORDEN, E. C. 2002. Anticancer activity of sodium stibogluconate in synergy with IFNs. *J Immunol,* 169**,** 5978-85.

1st Revision - Editorial Decision 25th Aug 2022

Dear Prof. Guarda,

Thank you for the submission of your revised manuscript to our editorial offices. I have now received the reports from the two referees that I asked to re-evaluate your study, you will find below. As you will see, both referees now support the publication of your study. Please go through the manuscript text to identify parts of the manuscript where clarity can be improved (see the comment of referee #1). Going through your point-by-point-response, I consider the points of referee #3 as adequately addressed.

Before proceeding with formal acceptance, I have these editorial requests I also you to address in a final revised manuscript:

- Please add up to 5 key words to the title page.

- Please add to the 'Disclosure and Competing Interests Statement' that all the other authors declare no competing interests and have no disclosures (if this is the case) or declare competing interests also for the other authors.

- We now use CRediT to specify the contributions of each author in the journal submission system. CRediT replaces the author contribution section. Please use the free text box to provide more detailed descriptions. Thus, please remove the author contributions section from the manuscript text file. See also guide to authors: https://www.embopress.org/page/journal/14693178/authorguide#authorshipguidelines

- There seems to be no separate callout for Fig. EV2E. Moreover, Fig. 1F is called out before Fig. 1E. Please make sure that all figure panels are called out separately and sequentially (or change the order of the panels in the figures).

- Please remove the referee token from the Data Availability section and make sure the dataset is public latest upon publication of the study.

- Please order the manuscript sections like this (using these names):
Title page - Abstract - Keywords - Introduction - Results & Discussion - Materials & Methods - DAS - Acknowledgements - Disclosure and Competing Interests Statement - References - Figure legends - Expanded View Figure legends

- Please make sure that the number "n" for how many independent experiments were performed, their nature (biological versus technical replicates), the bars and error bars (e.g. SEM, SD) and the test used to calculate p-values is indicated in the respective figure legends (main, EV and Appendix figures), and that statistical testing has been done where applicable. Please avoid phrases like 'independent experiment', but clearly state if these were biological or technical replicates. Please add complete statistical testing to all diagrams (main, EV and Appendix figures). Please also indicate (e.g. with n.s.) if testing was performed, but the differences are not significant. In case n=2, please show the data as separate datapoints without error bars and statistics.

- Please format the reference list according to our reference format:
http://www.embopress.org/page/journal/14693178/authorguide#referencesformat

- Thanks for providing source data. Please submit this as one PDF file per figure.

- Table EV1 is a dataset. Please name this Dataset EV1 and add its legend on the first TAB of the excel file. Finally, please remove the legend from the manuscript text file.

- Finally, please find attached a word file of the manuscript text (provided by our publisher) with changes we ask you to include in your final manuscript text, and some queries, we ask you to address. Please provide your final manuscript file (using the attached file as basis) with track changes, in order that we can see any modifications done.

In addition, I would need from you:
- a short, two-sentence summary of the manuscript (not more than 35 words).
- two to four short bullet points highlighting the key findings of your study (two lines each).
- a schematic summary figure (in jpeg or tiff format with the exact width of 550 pixels and a height of not more than 400 pixels) that can be used as a visual synopsis on our website.

I look forward to seeing the final revised version of your manuscript when it is ready. Please let me know if you have questions regarding the revision.

Please use this link to submit your revision: https://embor.msubmit.net/cgi-bin/main.plex

Best,

Achim Breiling
Senior Editor
EMBO Reports

Referee #1:

The authors have addressed the majority of my concerns. They also revised the manuscript to provide a more accurate, clear and balanced discussion of their results. Overall, the paper has been substantially improved, despite that some suggested experiments were not performed due to COVID related restrictions and ethical considerations. The clarity of the writing still has room for improvement.

Referee #2:

The revised version of this manuscript is substantially improved in all key points I had raised:

Figures have been amended as suggested i.e. axis labels reworked, details on the gating strategy included in Figure legend.
The introduction and discussion sections provide additional clarity e.g. on earlier results. Specifically, the discussion of differences between CD4Cre Ptpn6 fl/fl and CD4Cre Ptpn11fl/fl mice in terms of anticancer responses and therapeutic effects upon PD-1 blockade are very helpful. The divergent effects of Shp-1 and Shp-2 deletion became far more clear.
The discussion has been modified to acknowledge limitations of the study and clarify certain ambiguities.
Additional data sets have been included as requested (as an example: tumor volume curves as well as a detailed analysis of CD4 T cells).

In summary, the authors have provided a detailed and thorough response to all comments and have successfully addressed all my major concerns. I really enjoyed reading the revised paper!

The authors have addressed all minor editorial requests.

2nd Revision - Editorial Decision 8th Sep 2022

Prof. Greta Guarda
Institute for Research in Biomedicine
Via Chiesa 5
Bellinzona, Ticino 6500
Switzerland

Dear Prof. Guarda,

I am very pleased to accept your manuscript for publication in the next available issue of EMBO reports. Thank you for your contribution to our journal.

At the end of this email I include important information about how to proceed. Please ensure that you take the time to read the information and complete and return the necessary forms to allow us to publish your manuscript as quickly as possible.

As part of the EMBO publication's Transparent Editorial Process, EMBO reports publishes online a Review Process File to accompany accepted manuscripts. As you are aware, this File will be published in conjunction with your paper and will include the referee reports, your point-by-point response and all pertinent correspondence relating to the manuscript.

If you do NOT want this File to be published, please inform the editorial office within 2 days, if you have not done so already, otherwise the File will be published by default [contact: emboreports@embo.org]. If you do opt out, the Review Process File link will point to the following statement: "No Review Process File is available with this article, as the authors have chosen not to make the review process public in this case."

Should you be planning a Press Release on your article, please get in contact with emboreports@wiley.com as early as possible, in order to coordinate publication and release dates.

Thank you again for your contribution to EMBO reports and congratulations on a successful publication. Please consider us again in the future for your most exciting work.

Yours sincerely,

Achim Breiling
Senior Editor
EMBO Reports

****

THINGS TO DO NOW:

Please note that you will be contacted by Wiley Author Services to complete licensing and payment information. The required 'Page Charges Authorization Form' is available here: https://www.embopress.org/pb-assets/embo-site/er_apc.pdf - please download and complete the form and return to embopressproduction@wiley.com

You will receive proofs by e-mail approximately 2-3 weeks after all relevant files have been sent to our Production Office; you should return your corrections within 2 days of receiving the proofs.

Please inform us if there is likely to be any difficulty in reaching you at the above address at that time. Failure to meet our deadlines may result in a delay of publication, or publication without your corrections.

All further communications concerning your paper should quote reference number EMBOR-2022-55399V3 and be addressed to emboreports@wiley.com.

Should you be planning a Press Release on your article, please get in contact with emboreports@wiley.com as early as possible, in order to coordinate publication and release dates.

EMBO Press Author Checklist

Corresponding Author Name: Greta Guarda	
Journal Submitted to: EMBO Reports	
Manuscript Number: EMBOR-2022-55399V2	

USEFUL LINKS FOR COMPLETING THIS FORM
The EMBO Journal - Author Guidelines
EMBO Reports - Author Guidelines
Molecular Systems Biology - Author Guidelines
EMBO Molecular Medicine - Author Guidelines

Reporting Checklist for Life Science Articles (updated January 2022)
This checklist is adapted from Materials Design Analysis Reporting (MDAR) Checklist for Authors. MDAR establishes a minimum set of requirements in transparent reporting in the life sciences (see Statement of Task: 10.31222/osf.io/9sm4x). Please follow the journal's guidelines in preparing your manuscript.
Please note that a copy of this checklist will be published alongside your article.

Abridged guidelines for figures
1. Data
The data shown in figures should satisfy the following conditions:
- the data were obtained and processed according to the field's best practice and are presented to reflect the results of the experiments in an accurate and unbiased manner.
- ideally, figure panels should include only measurements that are directly comparable to each other and obtained with the same assay.
- plots include clearly labeled error bars for independent experiments and sample sizes. Unless justified, error bars should not be shown for technical replicates.
- if n<5, the individual data points from each experiment should be plotted. Any statistical test employed should be justified.
- Source Data should be included to report the data underlying figures according to the guidelines set out in the authorship guidelines on Data Presentation.

2. Captions
Each figure caption should contain the following information, for each panel where they are relevant:
- a specification of the experimental system investigated (eg cell line, species name).
- the assay(s) and method(s) used to carry out the reported observations and measurements.
- an explicit mention of the biological and chemical entity(ies) that are being measured.
- an explicit mention of the biological and chemical entity(ies) that are altered/varied/perturbed in a controlled manner.
- the exact sample size (n) for each experimental group/condition, given as a number, not a range;
- a description of the sample collection allowing the reader to understand whether the samples represent technical or biological replicates (including how many animals, litters, cultures, etc.).
- a statement of how many times the experiment shown was independently replicated in the laboratory.
- definitions of statistical methods and measures:

  - common tests, such as t-test (please specify whether paired vs. unpaired), simple χ2 tests, Wilcoxon and Mann-Whitney tests, can be unambiguously identified by name only, but more complex techniques should be described in the methods section;
  - are tests one-sided or two-sided?
  - are there adjustments for multiple comparisons?
  - exact statistical test results, e.g., P values = x but not P values < x;
  - definition of 'center values' as median or average;
  - definition of error bars as s.d. or s.e.m.

Please complete ALL of the questions below.
Select "Not Applicable" only when the requested information is not relevant for your study.

Materials

Newly Created Materials	Information included in the manuscript?	In which section is the information available? (Reagents and Tools Table, Materials and Methods, Figures, Data Availability Section)
New materials and reagents need to be available; do any restrictions apply?	Not Applicable	

Antibodies	Information included in the manuscript?	In which section is the information available? (Reagents and Tools Table, Materials and Methods, Figures, Data Availability Section)
For **antibodies** provide the following information:<br>- Commercial antibodies: RRID (if possible) or supplier name, catalogue number and or/clone number<br>- Non-commercial: RRID or citation	Not Applicable	In the material and methods sections the clones are stated for each antibody used.

DNA and RNA sequences	Information included in the manuscript?	In which section is the information available? (Reagents and Tools Table, Materials and Methods, Figures, Data Availability Section)
**Short novel DNA or RNA including primers, probes**: provide the sequences.	Not Applicable	

Cell materials	Information included in the manuscript?	In which section is the information available? (Reagents and Tools Table, Materials and Methods, Figures, Data Availability Section)
**Cell lines:** Provide species information, strain. Provide accession number in repository **OR** supplier name, catalog number, clone number, and/**OR** RRID.	Not Applicable	MC38 cell line was used which is a well established cell line for mouse anticancer research.
**Primary cultures:** Provide species, strain, sex of origin, genetic modification status.	Yes	These informations are provided, but for sex, as both were interchangeably used. For more information please check the materials and methods section.
Report if the cell lines were recently **authenticated** (e.g., by STR profiling) and tested for mycoplasma contamination.	Not Applicable	We tested the cell lines used for the experiments for mycoplasma in the lab.

Experimental animals	Information included in the manuscript?	In which section is the information available? (Reagents and Tools Table, Materials and Methods, Figures, Data Availability Section)
**Laboratory animals or Model organisms:** Provide species, strain, sex, age, genetic modification status. Provide accession number in repository **OR** supplier name, catalog number, clone number, **OR** RRID.	Yes	All relevant information can be found in materials and methods. With regards to sex, both were equally used.
**Animal observed in or captured from the field:** Provide species, sex, and age where possible.	Not Applicable	
Please detail **housing and husbandry conditions**.	Yes	Mice were maintained and kept in accordance to the best pratice of both countries involved. Additional information can be found in the materials and methods section.

Plants and microbes	Information included in the manuscript?	In which section is the information available? (Reagents and Tools Table, Materials and Methods, Figures, Data Availability Section)
**Plants:** provide species and strain, ecotype and cultivar where relevant, unique accession number if available, and source (including location for collected wild specimens).	Not Applicable	
**Microbes:** provide species and strain, unique accession number if available, and source.	Not Applicable	

Human research participants	Information included in the manuscript?	In which section is the information available? (Reagents and Tools Table, Materials and Methods, Figures, Data Availability Section)
If collected and within the bounds of privacy constraints report on age, sex and gender or ethnicity for all study participants.	Not Applicable	

Core facilities	Information included in the manuscript?	In which section is the information available? (Reagents and Tools Table, Materials and Methods, Figures, Data Availability Section)
If your work benefited from core facilities, was their service mentioned in the acknowledgments section?	Yes	Please see the acknowledgment section.

Design

Study protocol	Information included in the manuscript?	In which section is the information available? (Reagents and Tools Table, Materials and Methods, Figures, Data Availability Section)
If study protocol has been **pre-registered, provide DOI in the manuscript**. For clinical trials, provide the trial registration number **OR** cite DOI.	Not Applicable	
Report the **clinical trial registration number** (at ClinicalTrials.gov or equivalent), where applicable.	Not Applicable	

Laboratory protocol	Information included in the manuscript?	In which section is the information available? (Reagents and Tools Table, Materials and Methods, Figures, Data Availability Section)
Provide DOI OR other citation details if **external detailed step-by-step protocols** are available.	Not Applicable	

Experimental study design and statistics	Information included in the manuscript?	In which section is the information available? (Reagents and Tools Table, Materials and Methods, Figures, Data Availability Section)
Include a statement about **sample size** estimate even if no statistical methods were used.	Not Applicable	Information on sample size is provided in the Figure legends.
Were any steps taken to minimize the effects of subjective bias when allocating animals/samples to treatment (e.g. **randomization procedure**)? If yes, have they been described?	Not Applicable	
Include a statement about **blinding** even if no blinding was done.	Not Applicable	
Describe **inclusion/exclusion criteria** if samples or animals were excluded from the analysis. Were the criteria pre-established?<br><br>If sample or data points were omitted from analysis, report if this was due to attrition or intentional exclusion and provide justification.	Yes	No data exclusion was applied unless all quality controls included in the experiment were met or technical issues were present.
For every figure, are **statistical tests** justified as appropriate? Do the data meet the assumptions of the tests (e.g., normal distribution)? Describe any methods used to assess it. Is there an estimate of variation within each group of data? Is the variance similar between the groups that are being statistically compared?	Yes	We selected the appropriate tests considering the common standards in the field. For more information regarding statistical analysis please refer to figure legends and materials and methods.

Sample definition and in-laboratory replication	Information included in the manuscript?	In which section is the information available? (Reagents and Tools Table, Materials and Methods, Figures, Data Availability Section)
In the figure legends: state number of times the experiment was **replicated** in laboratory.	Yes	The relevant information can be found in the figure legends.
In the figure legends: define whether data describe **technical or biological replicates**.	Yes	The relevant information can be found in the figure legends.

Ethics

Ethics	Information included in the manuscript?	In which section is the information available? (Reagents and Tools Table, Materials and Methods, Figures, Data Availability Section)
Studies involving **human participants**: State details of **authority granting ethics approval** (IRB or equivalent committee(s), provide reference number for approval.	Not Applicable	
Studies involving **human participants**: Include a statement confirming that **informed consent** was obtained from all subjects and that the experiments conformed to the principles set out in the WMA Declaration of Helsinki and the Department of Health and Human Services Belmont Report.	Not Applicable	
Studies involving **human participants:** For publication of **patient photos**, include a statement confirming that consent to publish was obtained.	Not Applicable	
Studies involving experimental **animals**: State details of **authority granting ethics approva**l (IRB or equivalent committee(s), provide reference number for approval. Include a statement of compliance with ethical regulations.	Yes	This information can be found in the materials and methods section under "Mice".
Studies involving **specimen and field samples:** State if relevant **permits** obtained, provide details of authority approving study; if none were required, explain why.	Not Applicable	

Dual Use Research of Concern (DURC)	Information included in the manuscript?	In which section is the information available? (Reagents and Tools Table, Materials and Methods, Figures, Data Availability Section)
Could your study fall under dual use research restrictions? Please check biosecurity documents and list of **select agents and toxins** (CDC): https://www.selectagents.gov/sat/list.htm	Not Applicable	
If you used a select agent, is the security level of the lab appropriate and reported in the manuscript?	Not Applicable	
If a study is subject to dual use research of concern regulations, is the name of the **authority granting approval and reference number** for the regulatory approval provided in the manuscript?	Not Applicable	

Reporting

The MDAR framework recommends adoption of discipline-specific guidelines, established and endorsed through community initiatives. Journals have their own policy about requiring specific guidelines and recommendations to complement MDAR.

Adherence to community standards	Information included in the manuscript?	In which section is the information available? (Reagents and Tools Table, Materials and Methods, Figures, Data Availability Section)
State if relevant guidelines or checklists (e.g., **ICMJE, MIBBI, ARRIVE, PRISMA**) have been followed or provided.	Not Applicable	
For **tumor marker prognostic studies,** we recommend that you follow the **REMARK** reporting guidelines (see link list at top right). See author guidelines, under 'Reporting Guidelines'. Please confirm you have followed these guidelines.	Not Applicable	
For **phase II and III randomized controlled trials**, please refer to the **CONSORT** flow diagram (see link list at top right) and submit the CONSORT checklist (see link list at top right) with your submission. See author guidelines, under 'Reporting Guidelines'. Please confirm you have submitted this list.	Not Applicable	

Data Availability

Data availability	Information included in the manuscript?	In which section is the information available? (Reagents and Tools Table, Materials and Methods, Figures, Data Availability Section)
Have **primary datasets** been deposited according to the journal's guidelines (see 'Data Deposition' section) and the respective accession numbers provided in the Data Availability Section?	Yes	Please see data availability section.
Were **human clinical and genomic datasets** deposited in a public access-controlled repository in accordance to ethical obligations to the patients and to the applicable consent agreement?	Not Applicable	
Are **computational models** that are central and integral to a study available without restrictions in a machine-readable form? Were the relevant accession numbers or links provided?	Not Applicable	
If publicly available data were reused, provide the respective **data citations in the reference list**.	Not Applicable	
